# An Innovative Sprayable Mg-MOF Hydrogel Enhances Healing of Chronic Wounds with Multifaceted Pathologies in a Porcine Model

**DOI:** 10.1021/acsnano.6c05950

**Published:** 2026-07-29

**Authors:** Shunxiang Xu, Kexin Zhang, Hongwei Shao, Qinyu Tian, Yuantao Zhang, Jiankun Xu, Congqin Ning, Ling Qin

**Affiliations:** † Musculoskeletal Research Laboratory, Department of Orthopaedics & Traumatology, Faculty of Medicine, 26451 The Chinese University of Hong Kong, New Territories, Hong Kong SAR 999077, P. R. China; ‡ Innovative Orthopaedic Biomaterial and Drug Translational Research Laboratory, Li Ka Shing Institute of Health Sciences, Prince of Wales Hospital, The Chinese University of Hong Kong, New Territories, Hong Kong SAR 999077, P. R. China; § The Education Ministry Key Lab of Resource Chemistry and Shanghai Frontiers Science Center of Biomimetic Catalysis and Shanghai Engineering Research Center of Green Energy Chemical Engineering, 12544 Shanghai Normal University, Shanghai 200234, P. R. China

**Keywords:** Mg-ZIF-8, tea polyphenol, sprayable hydrogel, chronic wound healing, porcine model

## Abstract

Chronic wounds are characterized by unsatisfactory clinical outcomes due to persistent inflammation, excessive oxidative stress, and impaired neurovascular function, which conventional dressings cannot adequately address. This study presents a promising strategy for sequentially reversing these multifactorial wound conditions. We engineered a sprayable Mg-ZIF-8@TP@Alg hydrogel by integrating a Mg–Zn bimetallic metal–organic framework (Mg-ZIF-8) with tea polyphenol (TP), achieving differential release and pH-responsive therapeutic kinetics. *In vitro* assays demonstrated that Mg-ZIF-8@TP exerted synergistic ROS-scavenging activity, promoted M2 macrophage polarization, and enhanced angiogenic and neurogenic outcomes. These effects are attributed to TP-mediated attenuation of oxidative stress, together with activation of Mg^2+^/Zn^2+^-driven cellular behaviors. In a clinically relevant porcine model, the Mg-ZIF-8@TP@Alg hydrogel significantly accelerated wound healing. It sequentially remodelled the immune microenvironment by decreasing pro-inflammatory iNOS^+^ cells and increasing reparative CD163^+^ macrophages. Moreover, it enhanced CD31^+^ vascular neogenesis and β3-tubulin^+^ peripheral nerve regeneration. Transcriptomic analysis revealed that the hydrogel orchestrated chronic wound repair by modulating key pathways associated with ion transport, immune response suppression, and skin development. Overall, this work presents an innovative sprayable Mg-MOF hydrogel with synergistic, stage-specific therapeutic functionality, offering a potential strategy for chronic wound management.

## Graphical abstract

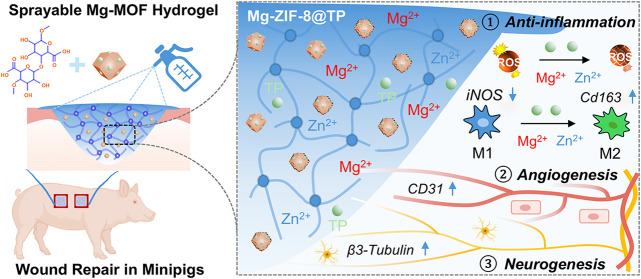

## Introduction

Chronic wounds, such as diabetic ulcers and pressure sores, affect millions of patients worldwide and impose substantial economic and social burdens. Unlike acute wounds that follow a well-orchestrated healing cascade of inflammation, proliferation, and remodelling, chronic wounds are trapped in a persistent inflammatory phase driven by interconnected multifaceted pathologies: dysregulated immune responses with M1 macrophage polarization, excessive reactive oxygen species (ROS)-mediated oxidative stress, and impaired neurovascular regeneration.^1,2^ The disrupted crosstalk between immune, vascular, and neuronal systems further amplifies this pathological cycle, leading to nonhealing tissue defects and poor clinical outcomes.^3^ Current clinical dressings are predominantly passive, providing only moisture retention and physical barrier protection, whereas emerging bioactive materials often fail to deliver stage-specific, sequential therapy that adapts to the dynamic pathological changes in chronic wounds.^4,5^ This critical gap necessitates the design of multifunctional biomaterials that can simultaneously reverse oxidative stress, reprogram the immune microenvironment, and promote neurovascular regeneration in a temporally coordinated manner.

In recent years, bioactive materials that can interact dynamically with the wound microenvironment have attracted considerable attention. Among them, metal–organic frameworks (MOFs) have emerged as promising platforms for drug delivery and tissue engineering, attributed to their high surface area and potential for degradability.^6,7^ Zeolitic imidazolate framework-8 (ZIF-8), composed of zinc ions and 2-methylimidazole, demonstrates excellent biocompatibility, pH-responsive degradation, and efficient drug-loading capability.^8,9^ However, the intrinsic bioactivity of pure ZIF-8 is limited for modulating chronic inflammation and driving neurovascular regeneration, hindering its standalone therapeutic efficacy. Magnesium ions (Mg^2+^) are well-recognized bioactive regulators that play pivotal roles in angiogenesis, nerve repair, and tissue regeneration.^10,11^ Mg-based biomaterials have even received regulatory approval for bone repair.^12,13^ Despite this, commercial Mg-based dressings for soft-tissue wound repair are still in their early stages. Doping Mg^2+^ into the ZIF-8 lattice to form Mg–Zn bimetallic MOF (Mg-ZIF-8) is therefore a rational strategy to enhance the pro-angiogenic and neurogenic potential of ZIF-8 while preserving its structural advantages for drug delivery.^14^

Natural polyphenols, such as tea polyphenol (TP), possess potent ROS-scavenging and anti-inflammatory properties, capable of suppressing pro-inflammatory cytokine production and promoting M2 macrophage polarization, thereby alleviating the hostile microenvironment of chronic wounds.^15,16^ Nevertheless, TP lacks direct capacity to drive robust tissue repair and neurovascular regeneration, limiting its clinical therapeutic effect.^17^ We hypothesized that integrating TP into Mg-ZIF-8 nanoparticles would yield a composite with synergistic and sequential biofunctions: TP would provide rapid antioxidant and anti-inflammatory effects, while sustained release of Mg^2+^ and Zn^2+^ from Mg-ZIF-8 would drive prolonged neurovascular regeneration.^18^ More importantly, the pH-responsive degradation of Mg-ZIF-8 and the differential release kinetics of TP (fast release) and Mg^2+^/Zn^2+^ (sustained release) would enable the material to mimic the natural wound-healing cascade.

In this study, we synthesized Mg-ZIF-8 nanoparticles and loaded them with TP to create Mg-ZIF-8@TP. This composite was then homogeneously incorporated into an alginate matrix to engineer a sprayable Mg-ZIF-8@TP@alginate (Alg) hydrogel. We systematically characterized the material’s physicochemical properties, drug and ion release profiles, and *in vitro* bioactivities, including antioxidant, immunomodulatory, angiogenic, and neurogenic effects. The therapeutic efficacy and underlying mechanisms were further investigated in a clinically relevant chronic wound model in Bama minipigs, a large-animal model with skin structure and physiology closely resembling those of humans.^19^ RNA sequencing was employed to elucidate the molecular pathways regulating the sequential healing effect. Overall, this work presents a stimulus-responsive multifunctional hydrogel dressing that enables temporal immunomodulation and coupled immune-angiogenic-neuronic repair for chronic wounds (Scheme 1).

**Scheme 1:**
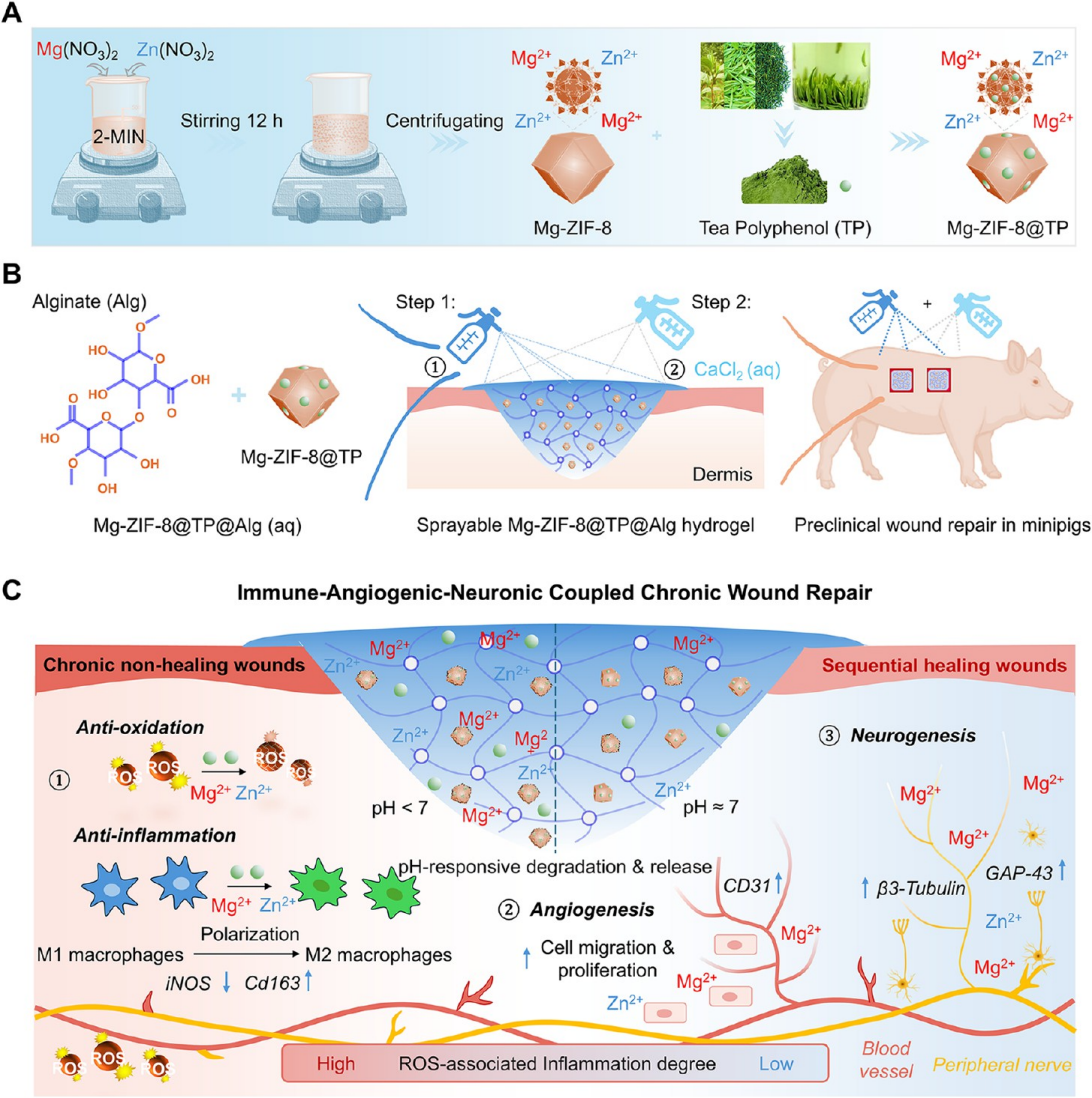
Illustration of Preparation and Underlying Mechanisms of Mg-ZIF-8@TP@Alg Sprayable Hydrogel for Chronic Wound Repair in a Porcine Model^a^

a (A, B) Synthesis of sprayable hydrogel: Mg-ZIF-8 nanoparticles are synthesized, loaded with tea polyphenol, and then mixed with alginate to prepare the Mg-ZIF-8@TP@Alg hydrogel, which can be smoothly sprayed onto the wound area. (C) pH-responsive degradation and immune-angiogenic-neuronic coupled repair: The Mg-ZIF-8@TP@Alg hydrogel demonstrates pH-responsive degradation *in vivo*. The released TP, Mg^2+^, and Zn^2+^ sequentially contribute to antioxidant, anti-inflammatory, pro-angiogenic, and neurogenic effects, thereby synergistically accelerating chronic wound healing in minipigs.

## Results and Discussion

### Synthesis and Characterization of Mg-ZIF-8 Nanoparticles

Mg-ZIF-8 nanoparticles were synthesized via a facile one-pot method by coordination with 2-methylimidazole and Zn^2+^ (Figure 1A).^20,21^ X-ray diffraction (XRD) analysis confirmed that Mg-ZIF-8 exhibited characteristic diffraction peaks consistent with the synthesized ZIF-8 and standard ZIF-8 crystal structure (CCDC-1429243), indicating that Mg^2+^ doping did not alter the crystal phase and basic framework of ZIF-8, a critical finding that ensures the retention of ZIF-8′s drug-loading and pH-responsive properties (Figure 1B). A high-angle annular dark-field (HAADF)-STEM image and elemental mapping confirmed the homogeneous distribution of C, N, Zn, and Mg within the nanoparticles, indicating successful Mg^2+^ incorporation (Figure 1C). Transmission electron microscopy (TEM) images revealed that Mg-ZIF-8 possessed a uniform dodecahedral morphology with an average particle size of approximately 50 nm (Figure 1D,E). The selected-area electron diffraction (SAED) pattern displayed diffuse rings (Figure 1F), indicating low crystallinity, which facilitates faster degradation and subsequent ion release in the biological microenvironment of chronic wounds.^22^

**Figure 1:**
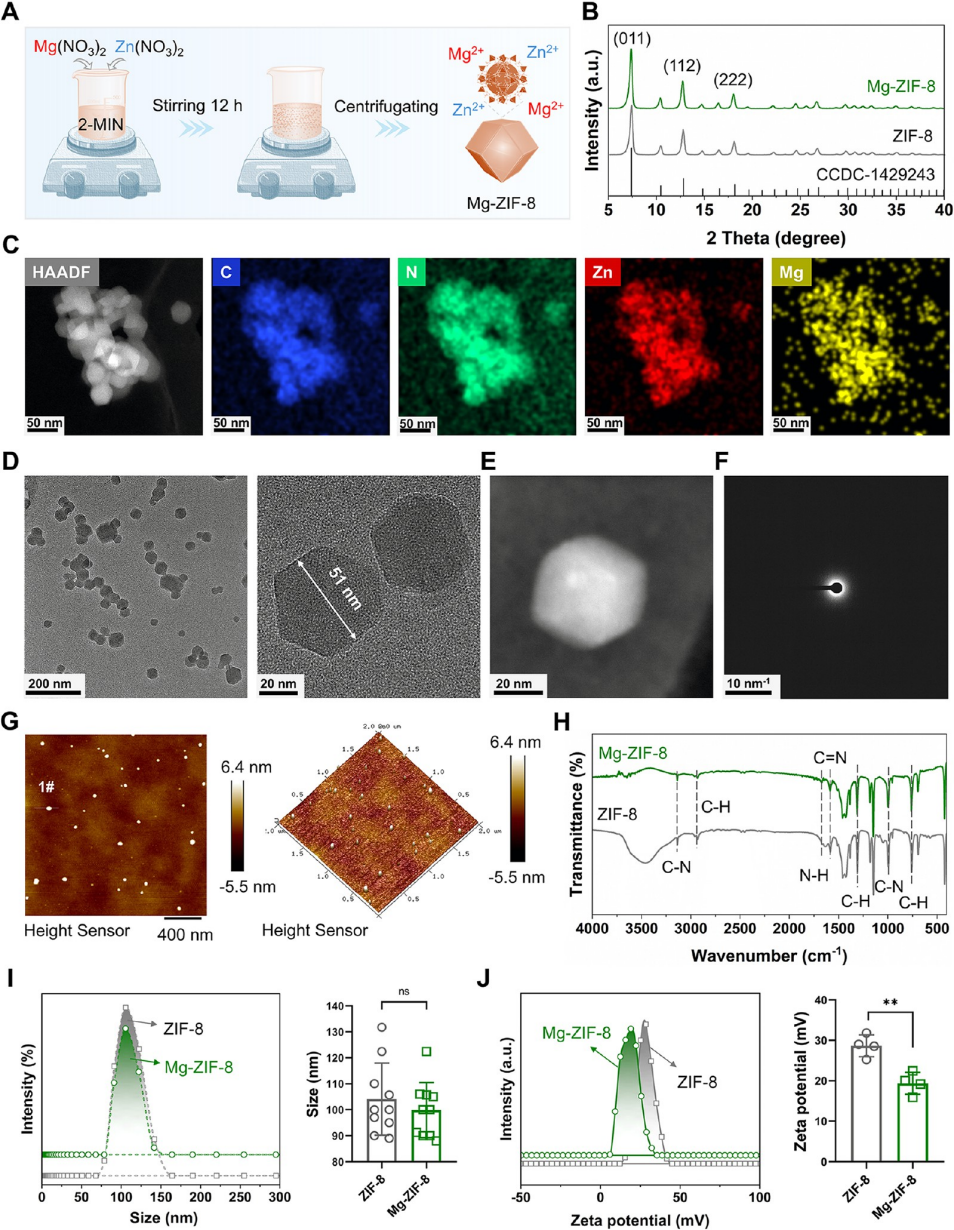
Synthesis and characterization of Mg-ZIF-8 nanoparticles. (A) Schematic diagram of the Mg-ZIF-8 synthesis process. (B) XRD patterns of synthesized Mg-ZIF-8 and ZIF-8 nanoparticles. (C) Representative HAADF image and element mappings (C, N, Zn, Mg) of Mg-ZIF-8. (D, E) Representative TEM images of Mg-ZIF-8 nanoparticles. (F) Representative SAED pattern of Mg-ZIF-8. (G) Representative AFM images of Mg-ZIF-8 in 2D and 3D views. (H) FTIR spectra of synthesized Mg-ZIF-8 and ZIF-8 nanoparticles. (I) Particle size distribution and related statistical analysis of synthesized Mg-ZIF-8 and ZIF-8 nanoparticles. (J) Zeta potential and corresponding statistical analysis.

Atomic force microscopy (AFM) images revealed the dodecahedral morphology of Mg-ZIF-8 nanoparticles with a nanoscale thickness (Figures 1G and S1A), and Fourier transform infrared (FTIR) spectroscopy confirmed the preservation of key ZIF-8 functional groups (C–H, C–N, C=N, N–H) in Mg-ZIF-8 (Figure 1H), ruling out structural damage during doping. Dynamic light scattering (DLS) results indicated that Mg-ZIF-8 had an average hydrodynamic size of 100 nm and a zeta potential of 20 mV, slightly smaller and less positively charged than pure ZIF-8 (105 nm, 28 mV) (Figure 1I,J), which may be attributed to the substitution of Zn^2+^ with Mg^2+^ and the formation of surface defect sites.^23^ The hydrodynamic size measured by DLS is larger than the actual particle size observed by TEM, primarily due to nanoparticle agglomeration. Collectively, these results demonstrate the successful synthesis of Mg–Zn bimetallic MOF (Mg-ZIF-8) nanoparticles with preserved ZIF-8 structural advantages and Mg^2+^-endowed bioactive potential, laying a solid foundation for subsequent drug loading and biofunctionalization.

### Preparation and Characterization of Mg-ZIF-8@TP and Composite Hydrogel

Mg-ZIF-8 nanoparticles were used as a carrier for TP loading (Figure 2A). TEM and AFM images confirmed that Mg-ZIF-8@TP nanoparticles retained the dodecahedral morphology of Mg-ZIF-8 with uniform dispersion (Figures 2B–C and S1B). XRD analysis showed no significant change in the crystal phase of Mg-ZIF-8 after TP loading (Figure 2D), indicating that the drug loading process did not disrupt the MOF framework. FTIR spectroscopy exhibited additional characteristic peaks of TP (−OH, C=O, C–O) in Mg-ZIF-8@TP (Figure 2E), directly confirming successful TP loading.^24^ DLS results showed that Mg-ZIF-8@TP had an average hydrodynamic size of 138 nm and a zeta potential of −15.6 mV (Figure 2F), a reverse in charge compared to Mg-ZIF-8 (20 mV), which was attributed to the dissociation of phenolic hydroxyl groups in TP and the release of H^+^ in aqueous solution; the negative zeta potential is beneficial for improving the biocompatibility and cellular uptake of the nanoparticles.^25,26^ TP was loaded into Mg-ZIF-8 via physical adsorption and chelation between the polyphenolic hydroxyl groups of TP and Mg^2+^ in the MOF, yielding an approximately 30% loading efficiency (Figures 2G and S2–S3), a value comparable to or higher than that of other ZIF-8-based drug delivery systems.^27^

**Figure 2:**
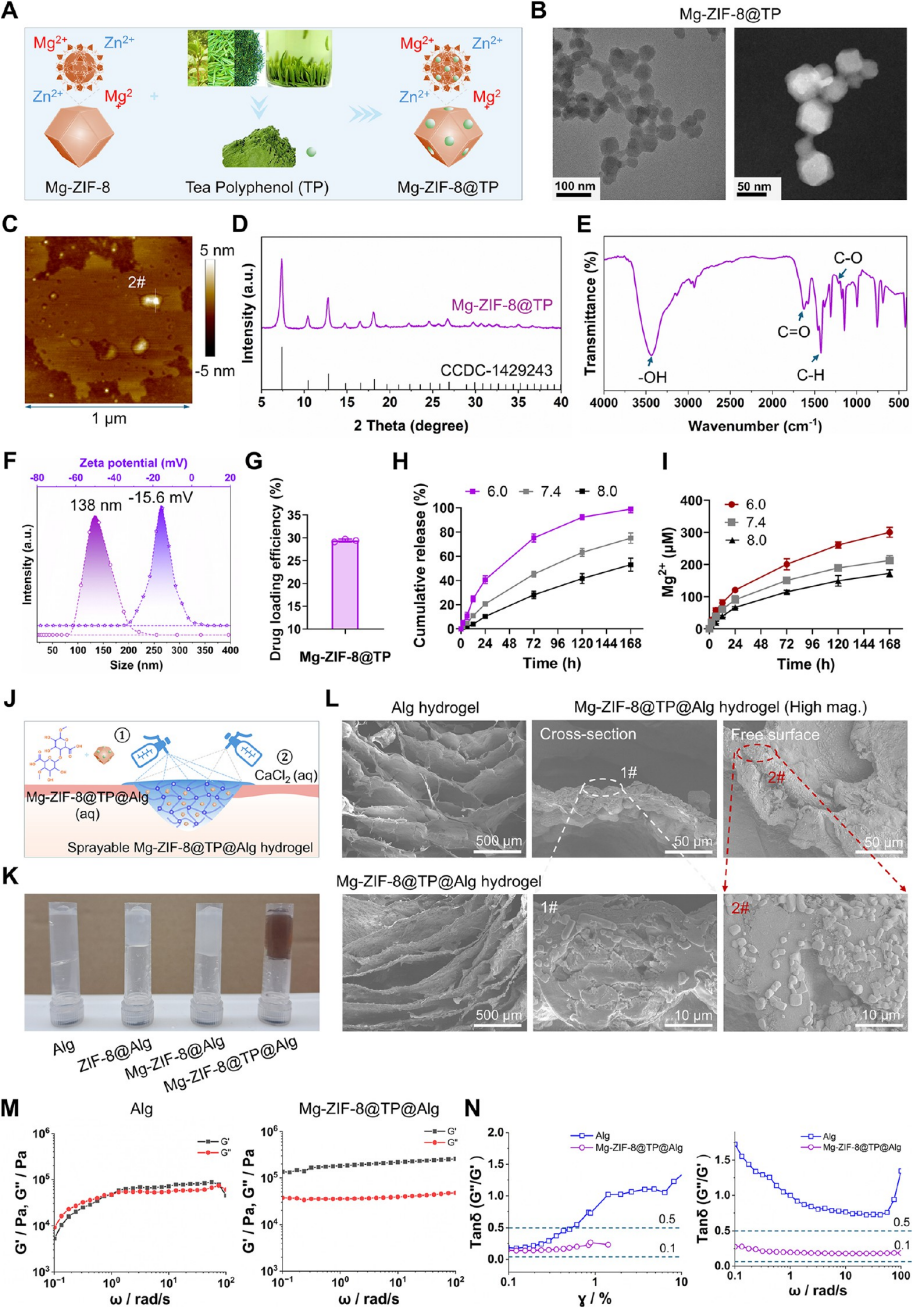
Synthesis and characterization of Mg-ZIF-8@TP nanoparticles and Mg-ZIF-8@TP@Alg composite hydrogel. (A) Schematic diagram of Mg-ZIF-8@TP nanoparticle synthesis process. (B) Representative TEM and STEM images of Mg-ZIF-8@TP nanoparticles. (C) Representative AFM image of Mg-ZIF-8@TP in 2D view. (D, E) XRD patterns and FTIR spectra of synthesized Mg-ZIF-8@TP nanoparticles. (F) Particle size distribution and Zeta potential of Mg-ZIF-8@TP. (G) Loading efficiency of Mg-ZIF-8 for the drug TP. (H) Cumulative release of TP from Mg-ZIF-8@TP nanoparticles under different pH conditions (pH = 6.0, 7.4, 8.0). (I) Cumulative release of Mg^2+^ from Mg-ZIF-8@TP nanoparticles under different pH conditions (pH = 6.0, 7.4, 8.0). (J, K) Schematic of preparation and results for sprayable composite hydrogels (Alg, ZIF-8@Alg, Mg-ZIF-8@Alg, Mg-ZIF-8@TP@Alg). (L) Representative SEM images of pure Alg and Mg-ZIF-8@TP@Alg composite hydrogel (1#: cross-section; 2#: free surface). (M) Frequency sweep curves (*G*′, *G*″ vs ω) of pure Alg and Mg-ZIF-8@TP@Alg composite hydrogel. (N) Loss tangent (tan δ = *G*″/*G*′) as a function of strain and frequency. Dashed lines indicate tan δ = 0.5 and 0.1.

*In vitro* drug and ion release assays were critical for validating the material’s sequential-release design. Considering chronic wound exudate is often alkaline, while the local microenvironment at the biofilm-tissue interface can become markedly acidic (pH 5.5–6.5) due to bacterial metabolism and pus accumulation, and the normal physiological environment is around 7.4, so three representative values were adopted (pH = 6.5, 7.4, 8.0).^28,29^ Notably, the release behavior exhibited a clear pH-dependence (Figure 2H). Under acidic conditions (pH 6.0), the cumulative release of TP reached ∼40% within 24 h and nearly 100% at 168 h, significantly exceeding that at pH 7.4 (∼70%) and pH 8.0 (∼55%). This quick-release formulation is essential for rapid ROS clearance and early stage inflammation resolution in chronic wound repair, while a sustained-release formulation ensures long-term suppression of oxidative stress. TEM images after 4 weeks of degradation in PBS confirmed the biodegradability of Mg-ZIF-8@TP, with significant structural fragmentation and size reduction (Figure S4A). Similarly, the release of both Mg^2+^ and Zn^2+^ ions followed the same pH-responsive trend, with pH 6.0 triggering the highest ionic release (Figures 2I and S4B). The burst release observed within the first 24 h allowed for immediate action, while the subsequent sustained release ensured prolonged therapeutic effects. Notably, Zn^2+^ release was more sensitive to pH changes, whereas the cumulative Mg^2+^ release reached approximately 300 μM, markedly higher than that of Zn^2+^ (∼70 μM). This difference arises from their distinct coordination environments: Zn^2+^ as the primary structural node of ZIF-8 is released with framework degradation, while Mg^2+^ occupies labile surface or defect sites and is preferentially released via ion exchange or surface detachment due to its weaker Mg–N/O bonds.^30,31^ The sustained, high-release of Mg^2+^ is a key design feature, as Mg^2+^ is the core bioactive ion that drives neurovascular regeneration in the middle stage of wound healing. This pH-responsive and sustained codelivery of TP and bioactive ions makes the hydrogel a promising smart dressing for accelerated wound healing.

To enhance practicality and ensure moisture retention in alignment with wet-healing theory, we used a commercial alginate matrix as the organic substrate.^32,33^ Mg-ZIF-8@TP nanoparticles were homogeneously incorporated into alginate to form the first part of a sprayable hydrogel, with the cross-linker (aqueous CaCl_2_ solution) as the second part (Figure 2J). After spraying the initial Mg-ZIF-8@TP@Alg solution, the composite hydrogel was effectively cross-linked by subsequently spraying the CaCl_2_ solution. As shown in Table S1, we conservatively estimated the *in situ* gelation time to be <5 s across all groups. As a result, we obtained pure Alginate (Alg) and composite hydrogels: ZIF-8@Alg, Mg-ZIF-8@Alg, and Mg-ZIF-8@TP@Alg, as shown in Figure 2K. Scanning electron microscopy (SEM) images showed that both pure Alg and Mg-ZIF-8@TP@Alg hydrogels possessed a porous microstructure (Figure 2L), which could facilitate the absorption of wound exudate, nutrient exchange, and cell adhesion and growth. Uniformly distributed Mg-ZIF-8@TP nanoparticles were observed in the cross-section and on the free surface, confirming the successful preparation of a sprayable, porous, and bioactive composite hydrogel that combines the moisture retention of alginate with the sequential biofunctions of Mg-ZIF-8@TP. Rheological results revealed that Mg-ZIF-8@TP@Alg composite hydrogel possesses significantly improved mechanical stability compared to pristine Alg. In strain sweep tests (Figure S5), pure Alg showed a narrow linear viscoelastic region (LVR, <0.1% strain), while the composite hydrogel exhibited a remarkably broadened LVR with stable storage modulus (*G*′) exceeding and loss modulus (*G*″) up to 10% strain. Frequency sweep measurements (Figure 2M) demonstrated that, unlike the frequency-dependent moduli of pure Alg, the composite displayed frequency-independent behavior with *G*′ consistently an order of magnitude higher than *G*″, indicating a robust cross-linked network. Furthermore, the loss tangent (tan δ) of the composite remained between 0.1 and 0.5 across all tested ranges (Figure 2N), confirming its predominantly elastic, solid-like network, in contrast to the viscous behavior (tan δ > 1) of pure Alg. Conformability, adhesion, filling capacity, and flexibility of Alg and Mg-ZIF-8@TP@Alg hydrogels on *ex vivo* porcine skin indicated that these values are comparable to those of wound dressings requiring flexibility and mechanical integrity (Figure S6).

### *In Vitro* Antioxidant and Immunomodulatory Activities

Chronic wounds are characterized by excessive ROS and a pro-inflammatory microenvironment dominated by M1 macrophages; reversing this pathological state is the first step toward effective wound healing.^34^ We first evaluated the cytocompatibility of Mg-ZIF-8 in RAW 264.7 macrophages. Cell counting kit-8 (CCK-8) assays and live/dead staining confirmed that Mg-ZIF-8 at concentrations up to 10 μg/mL promoted cell proliferation, while 50 μg/mL induced significant cytotoxicity (Figure 3A,B); thus, 10 μg/mL was selected for subsequent *in vitro* experiments.

**Figure 3:**
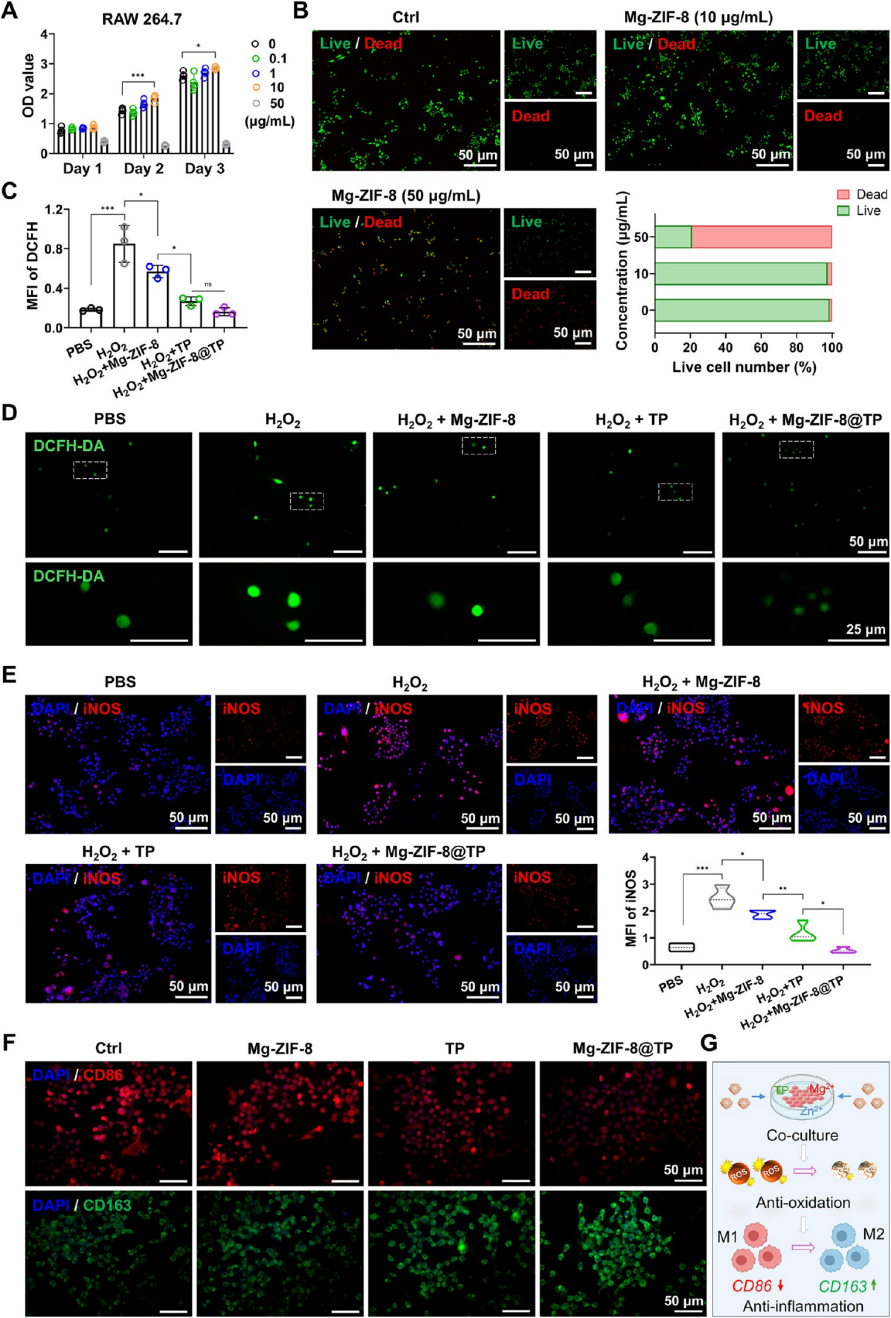
Evaluation of *in vitro* antioxidation and anti-inflammatory ability. (A) CCK-8 cell proliferation of RAW 264.7 macrophages with the treatment of various concentrations (0, 0.1, 1, 10, 50 μg/mL) of Mg-ZIF-8 nanoparticles for 1, 2, and 3 days. * *P* < 0.05, *** *P* < 0.001, by two-way ANOVA with Bonferroni *post hoc* test. (B) Representative live/dead staining images and statistical results of RAW 264.7 macrophages treated with Mg-ZIF-8 nanoparticles (0, 10, 50 μg/mL) for 2 days. (C–D) ROS levels detected by the DCFH-DA assay and statistical analysis of RAW 264.7 macrophages treated with PBS, H_2_O_2_, H_2_O_2_+Mg-ZIF-8 (10 μg/mL), H_2_O_2_+TP (3 μg/mL), and H_2_O_2_+Mg**-**ZIF-8@TP (13 μg/mL). * P **<**0.05, *** *P* < 0.001, by one-way ANOVA with Bonferroni *post hoc* test. (E) Representative iNOS staining images and statistical results of RAW 264.7 macrophages treated with PBS, H_2_O_2_, H_2_O_2_+Mg-ZIF-8, H_2_O_2_+TP, and H_2_O_2_+Mg-ZIF-8@TP for 2 days. * *P* < 0.05, ** *P* < 0.01, *** *P* < 0.001, by one**-**way ANOVA with Bonferroni *post hoc* test. (F) Representative IF staining images of M1 (CD86) and M2 (CD163) macrophage phenotypes. (G) *In vitro* antioxidation and anti-inflammation mechanism of Mg**-**ZIF-8@TP nanoparticles.

ROS-scavenging assays with the DCFH-DA probe showed that H_2_O_2_ stimulation significantly increased intracellular ROS levels in macrophages. Mg-ZIF-8 alone moderately reduced ROS levels, TP alone exhibited a stronger scavenging effect, and Mg-ZIF-8@TP nearly eliminated intracellular ROS (Figure 3C,D), demonstrating a synergistic antioxidant effect. This synergy is attributed to two mechanisms: TP directly scavenges ROS via its phenolic hydroxyl groups, while Mg^2+^ and Zn^2+^ indirectly modulate oxidative stress by protecting mitochondrial function in immune cells.^35,36^ Inducible nitric oxide synthase (iNOS), a key pro-inflammatory marker in M1 macrophages, was used to evaluate anti-inflammatory activity.^37^ Immunofluorescence staining showed that H_2_O_2_-induced iNOS expression was moderately reduced by Mg-ZIF-8, markedly attenuated by TP, and most significantly suppressed by Mg-ZIF-8@TP (Figure 3E), confirming the synergistic anti-inflammatory effect of the composite.

Macrophage polarization is a core regulatory target for reprogramming the immune microenvironment in chronic wounds. Immunofluorescence staining for M1 (Cluster of differentiation 86, CD86) and M2 (Cluster of differentiation 163, CD163) markers revealed that Mg-ZIF-8@TP treatment significantly downregulated CD86 expression and upregulated CD163 expression compared to Mg-ZIF-8 or TP alone (Figure 3F). These results confirm that the combined release of TP, Mg^2+^, and Zn^2+^ from Mg-ZIF-8@TP cooperatively mitigates oxidative stress and reprograms macrophage polarization from M1 to M2, thereby creating a pro-healing immune microenvironment (Figure 3G). TP plays a dominant role in the early suppression of pro-inflammatory and oxidative signaling, while Mg^2+^ and Zn^2+^ further enhance M2 polarization, thereby producing a synergistic effect that is far superior to single-component treatment and addresses the limitations of pure TP or MOFs in immune modulation.

### *In Vitro* Pro-Angiogenic Performance

Angiogenesis is essential for supplying oxygen and nutrients to the wound bed, and its impairment is a major cause of chronic wound nonhealing during the proliferative phase.^38^ We evaluated the pro-angiogenic potential of Mg-ZIF-8@TP using human umbilical vein endothelial cells (HUVECs), the primary cell type involved in vascular formation. Cytocompatibility assays confirmed that Mg-ZIF-8 at 10 μg/mL exhibited good biocompatibility with HUVECs, while 50 μg/mL induced cytotoxicity (Figure 4A,B). Compared with ZIF-8, Mg-ZIF-8 significantly stimulated blood vessel formation, validating the positive role of Mg^2+^ doping in angiogenesis (Figure S7). Under H_2_O_2_-induced oxidative stress, Mg-ZIF-8@TP exhibited superior ROS scavenging efficacy compared to Mg-ZIF-8 or TP alone (Figure 4C,D), which is critical for protecting endothelial cells from oxidative damage and restoring their biological functions. Scratch assays showed that H_2_O_2_ stimulation significantly inhibited HUVEC migration. Mg-ZIF-8 alone partially restored migration capacity, TP alone had a limited effect, and Mg-ZIF-8@TP synergistically promoted HUVEC migration to the greatest extent (Figure 4D,E). In tube formation assays, the gold standard for evaluating *in vitro* angiogenesis, Mg-ZIF-8@TP induced the formation of the most extensive and mature vascular networks (more meshes and branches) compared to all other groups (Figures 4F,G and S8). The synergistic pro-angiogenic effect of Mg-ZIF-8@TP arises from the coordination of TP-mediated ROS clearance and the activation of bioactive-ion (Mg^2+^ and Zn^2+^)-driven signaling pathways (Figure 4H). TP scavenges ROS to reduce endothelial cell damage and remove the oxidative stress barrier for angiogenesis. The released Mg^2+^ and Zn^2+^ engage the PI3K-AKT and VEGF pathways, respectively, as essential functional mediators, based on the observed rescue effects (Figure S9), which are consistent with the findings reported in the literature.^39,40^ This three-pronged mechanism addresses the limitation of single antioxidants or bioactive ions and peptides in angiogenesis and provides a robust pro-angiogenic stimulus for chronic wound repair.^41,42^

**Figure 4:**
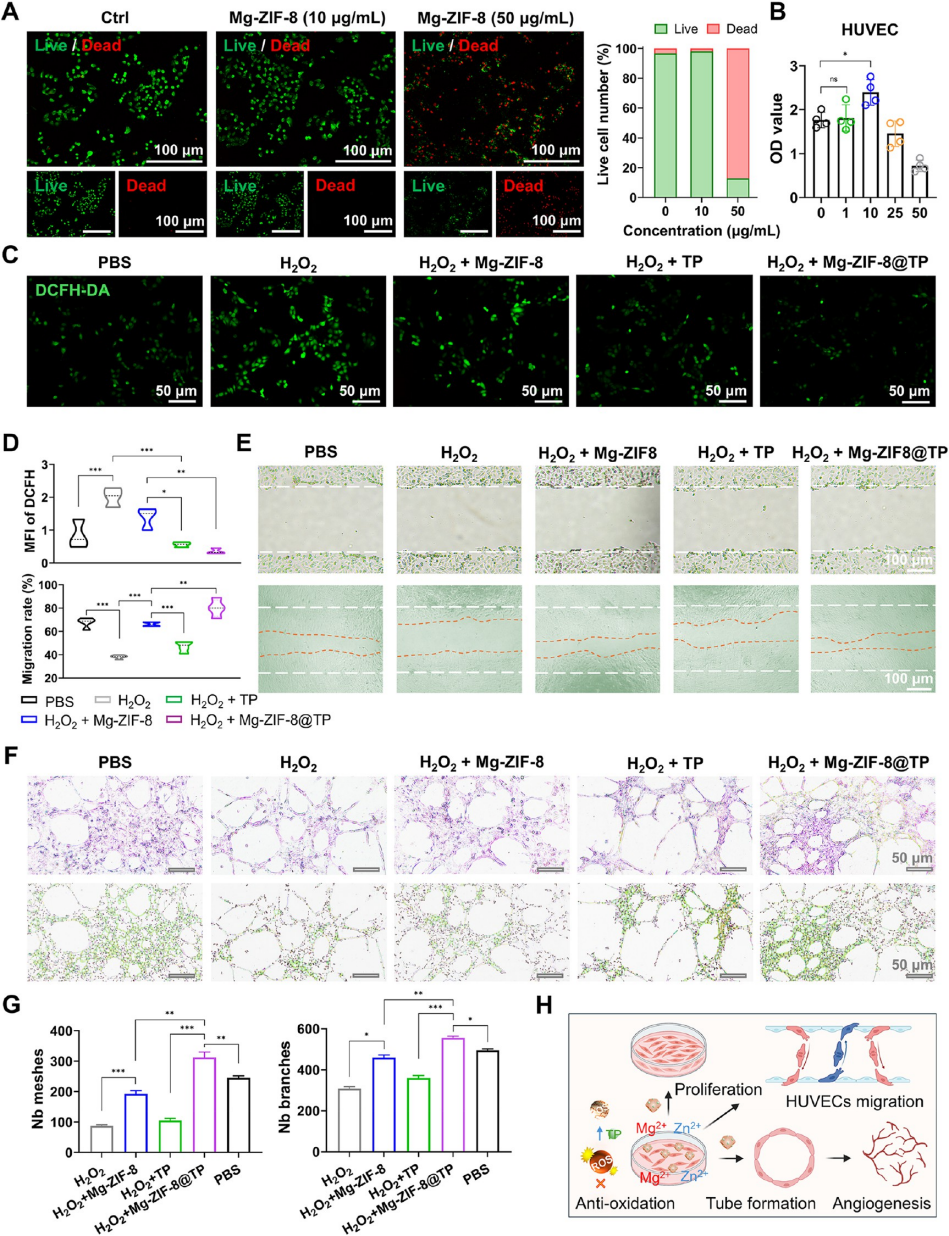
Evaluation of *in vitro* angiogenesis ability. (A) Representative live/dead staining images and statistical results of HUVEC treated with Mg-ZIF-8 nanoparticles (0, 10, 50 μg/mL) for 2 days. (B) CCK-8 cell viability assay of HUVECs treated with various concentrations of Mg-ZIF-8 (0, 1, 10, 25, 50 μg/mL) for 2 days. * *P* < 0.05, by one-way ANOVA with Bonferroni *post hoc* test. (C) ROS levels detected by the DCFH-DA assay of HUVEC treated with PBS, H_2_O_2_, H_2_O_2_+Mg-ZIF-8 (10 μg/mL), H_2_O_2_+TP (3 μg/mL), and H_2_O_2_+Mg-ZIF-8@TP (13 μg**/**mL). (D) Statistical analysis of DCFH mean fluorescent intensity (MFI) and migration rate. * *P* < 0.05, ** *P* < 0.01, *** *P* < 0.001, by one-way ANOVA with Bonferroni *post hoc* test. (E) Representative cell migration images of HUVEC treated with PBS, H_2_O_2_, H_2_O_2_+Mg-ZIF-8, H_2_O_2_+TP, and H_2_O_2_+Mg-ZIF-8@TP for 18 h. **(**F-G) Representative tube-formation images and statistical results of HUVECs treated with PBS, H_2_O_2_, H_2_O_2_+Mg-ZIF-8, H_2_O_2_ **+**TP, and H_2_O_2_+Mg-ZIF-8@TP for 6 h. * *P* < 0.05, ** *P* < 0.01, *** *P* **<**0.001, by one**-**way ANOVA with Bonferroni *post hoc* test. The first row shows the original data, and the second row shows the results from ImageJ analysis. (H) *In vitro* angiogenic mechanism of Mg-ZIF-8@TP nanoparticles.

### *In Vitro* Neurogenesis Performance

Peripheral nerve regeneration is closely linked to the quality of wound healing, and impaired neurite outgrowth leads to poor sensory recovery and delayed wound remodelling.^43^ We investigated the pro-neurogenic potential of Mg-ZIF-8@TP using primary Schwann cells (the main glial cells involved in peripheral nerve myelination) and dorsal root ganglion (DRG) neurons (a model for peripheral sensory neurons) isolated from 2-week-old rats (Figure 5A). CCK-8 assays confirmed that Mg-ZIF-8@TP at 10 μg/mL exhibited optimal biocompatibility with Schwann cells, supporting robust proliferation (Figure 5B).

**Figure 5:**
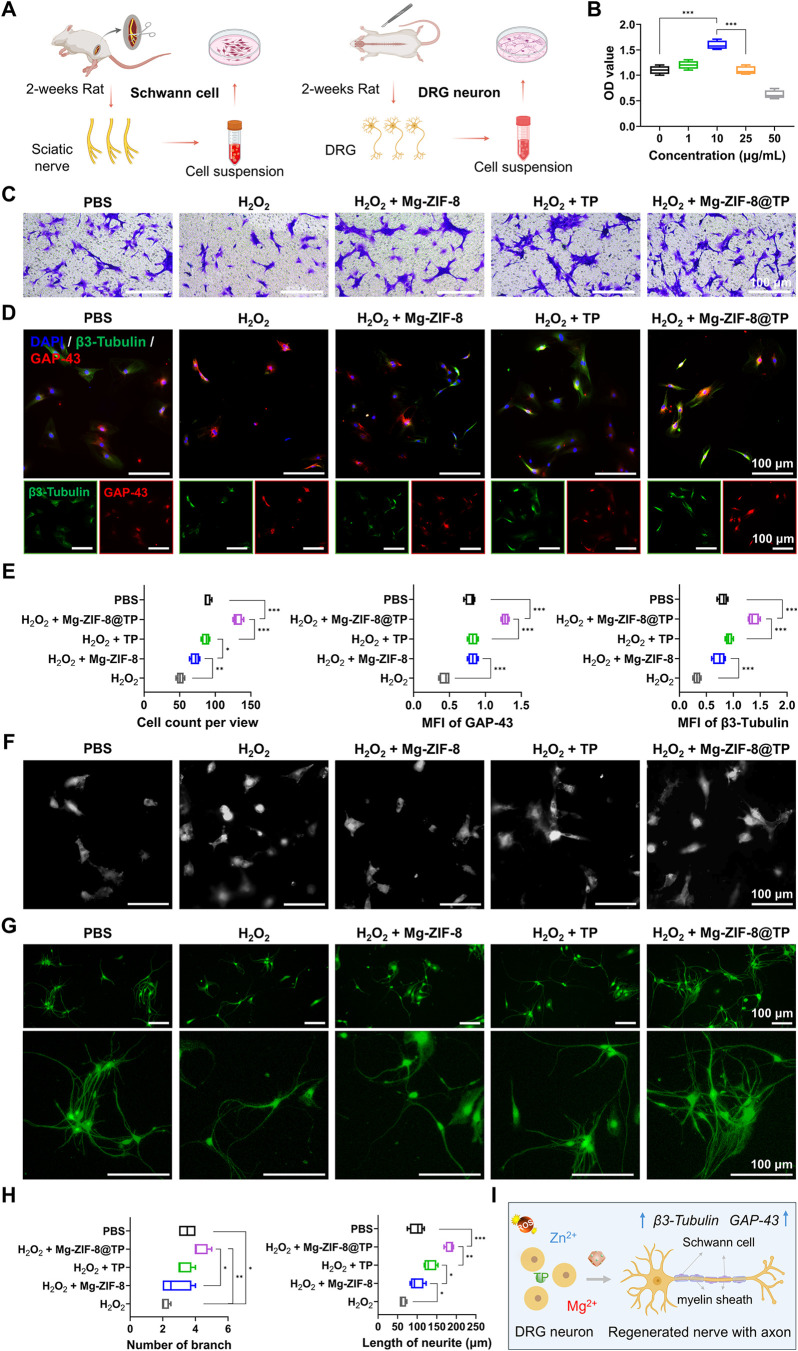
Evaluation of *in vitro* peripheral nerve regeneration. (A) Schematic illustration of the isolation process for primary Schwann cells (from sciatic nerves) and DRG neurons (from dorsal root ganglia) using 2-week-old rats. (B) CCK-8 cell viability of Schwann cells with the treatment of various Mg-ZIF-8 concentrations (0, 1, 10, 25, 50 μg/mL) for 2 days. *** *P* < 0.001, by one-way ANOVA with Bonferroni *post hoc* test. (C) Representative cell migration images of Schwann cells under different conditions for 24 h (crystal violet staining). (D) Representative IF images of Schwann cells under different conditions for 48 h (β-3 Tubulin, GAP-43). (E) Quantitative analysis of panel (C, D). * *P* < 0.05, ** *P* < 0.01, *** *P* < 0.001, by one-way ANOVA with Bonferroni *post hoc* test. (F–G) Morphological observations and axon regeneration of DRG neurons under different conditions over 3 days (GFP-transgenic rat-derived DRG). (H) Quantitative analysis of panel (G). * *P* < 0.05, ** *P* < 0.01, *** *P* < 0.001, by one-way ANOVA with Bonferroni *post hoc* test. (I)­Schematic diagram of peripheral nerve regeneration.

Under H_2_O_2_-induced oxidative stress, Schwann cell migration was significantly inhibited. Mg-ZIF-8 or TP alone partially reversed this inhibition, while Mg-ZIF-8@TP exerted the most potent pro-migratory effect (Figure 5C,E). Immunofluorescence staining for nerve regeneration markers showed that Mg-ZIF-8 significantly stimulated the expression of β3-Tubulin and GAP-43 more than ZIF-8, demonstrating the necessity of Mg^2+^ in promoting nerve regeneration (Figure S10). Furthermore, Mg-ZIF-8@TP significantly upregulated the expression of these proteins compared to single-component treatment or the H_2_O_2_ control group (Figure 5D,E), indicating that Mg-ZIF-8@TP promotes Schwann cell activation and myelination, a critical step for peripheral nerve regeneration. In DRG neurons, H_2_O_2_ stimulation led to marked neuronal atrophy and impaired neurite outgrowth. In contrast, Mg-ZIF-8@TP treatment restored neuronal morphology and promoted robust axon extension and branching (Figure 5F,G), with quantitative analysis confirming that Mg-ZIF-8@TP outperformed Mg-ZIF-8 or TP alone in axon length and branch number (Figure 5H).

The pro-neurogenic mechanism of Mg-ZIF-8@TP is a synergistic effect of TP-mediated oxidative stress relief and bioactive ions (Mg^2+^ and Zn^2+^)-driven neuronal activation (Figure 5I). TP scavenges intracellular ROS to alleviate oxidative damage to Schwann cells and DRG neurons, restoring their normal biological functions; Mg^2+^ and Zn^2+^ activate Schwann cell proliferation and migration to promote myelination, while driving DRG neuron axon outgrowth and branching via regulating neurotrophic factor signaling. Although other bioactive ions (Cu, Fe, Sr, etc.) and even rare-earth metal ions (Ce, Ru, etc.) doped into MOFs have been reported to regulate immunomodulatory and angiogenic properties, metal-MOFs that directly achieve neuroregulation remain limited.^44^ This work reports that Mg-MOF-based composites can promote peripheral nerve regeneration, expanding the biofunctional scope of MOF materials in soft tissue repair and nerve regeneration.

### Accelerated Wound Healing in a Minipig Chronic Wound Model

To evaluate the *in vivo* wound-healing efficacy of Mg-ZIF-8@TP, we incorporated the nanoparticles into a commercial alginate matrix to form corresponding composite hydrogels, leveraging the hydrogel’s moisture-retention capacity to facilitate moist wound healing. Given the high structural and functional similarity between porcine and human skin, we established an H_2_O_2_-induced ROS-overloaded chronic wound model in Bama minipigs, a clinically relevant large-animal model that recapitulates the pathological features of human chronic wounds (Figure 6A). Wounds were randomly divided into four groups: pure Alg (control), Mg-ZIF-8@Alg, TP@Alg, and Mg-ZIF-8@TP@Alg. Representative wound images at days 0, 3, 10, and 28 showed that the control group still had significant wound residues at day 28, while the three treatment groups achieved near-complete closure; notably, the Mg-ZIF-8@TP@Alg group exhibited the fastest wound closure rate and the most native skin-like tissue appearance (Figure 6B,C).

**Figure 6:**
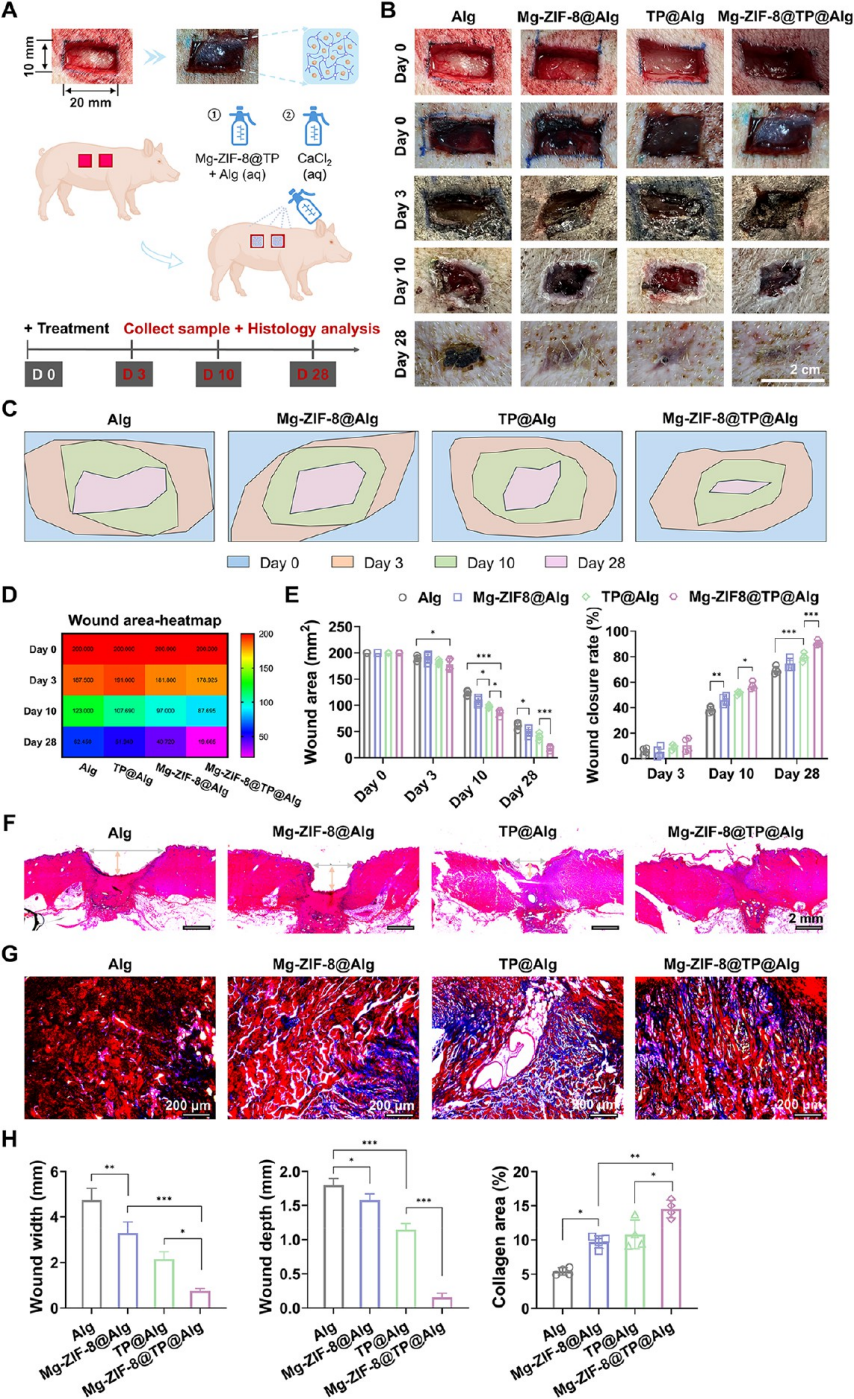
Accelerated wound repair in an H_2_O_2_-induced ROS-associated porcine model using Mg-ZIF-8@TP@Alg sprayable hydrogel. (A) Schematic diagram of the overall process for *in vivo* wound-healing evaluation. (B) Representative optical wound images after treating with Alg, Mg-ZIF-8@Alg, TP@Alg, and Mg-ZIF-8@TP@Alg for 3-, 10-, and 28-days postsurgery. (C) Schematic illustration of chronic wound closure process on 0-, 3-, 10-, and 28-day postwound. (D, E) Heatmap of residual wound area, statistical analysis of wound area, and closure rate at each time point. (F, G) Representative H&E and Masson staining images at 28 days postwound. * *P* < 0.05, ** *P* < 0.01, *** *P* < 0.001, by two-way ANOVA with Bonferroni *post hoc* test. (H) Statistical results for wound width, wound depth, and collagen area percentage at 28 days postsurgery. * *P* < 0.05, ** *P* < 0.01, *** *P* < 0.001, by one-way ANOVA with Bonferroni *post hoc* test.

Quantitative analysis of wound area reduction confirmed the superior efficacy of Mg-ZIF-8@TP@Alg. The healing rate at day 28 was 90.77 ± 1.96% for Mg-ZIF-8@TP@Alg, significantly higher than TP@Alg (79.64 ± 3.20%), Mg-ZIF-8@Alg (74.84 ± 3.73%), and the control group (Figure 6D,E). Histological analysis further validated the therapeutic effect. Hematoxylin and eosin (H&E) staining showed that the Mg-ZIF-8@TP@Alg group had complete wound closure with minimal wound width and depth (Figure 6F). Masson’s trichrome staining revealed significantly higher collagen deposition and more organized collagen fiber architecture in the Mg-ZIF-8@TP@Alg group (14.50 ± 1.29%) compared to the control (5.40 ± 0.53%), Mg-ZIF-8@Alg (9.69 ± 0.91%), and TP@Alg (10.78 ± 2.09%) groups (Figure 6G,H). Additionally, the healing indicators in the individual TP@Alg group were superior to those in the Mg-ZIF-8@Alg group, indicating the importance of early inflammatory clearance and immune regulation for sequential healing. These *in vivo* results confirm that the sequential therapeutic strategy of Mg-ZIF-8@TP@Alg hydrogel is far superior to single-component treatment. TP rapidly resolves inflammation in the early stages, thereby shortening the inflammatory phase, while the released bioactive ions (Mg^2+^ and Zn^2+^) sustainably promote tissue regeneration in the middle and late stages, thereby enhancing the quality of healing. This temporal coordination breaks the pathological cycle of chronic wounds and achieves robust, orderly tissue repair.

### Mg-ZIF-8@TP@Alg Hydrogel Reversed Multifaceted Pathologies in Porcine

The core mechanism by which the Mg-ZIF-8@TP@Alg hydrogel promotes chronic wound healing lies in its ability to reverse multifaceted pathologies and restore critical crosstalk among the immune, vascular, and neuronal systems. This multifactorial, system-level therapeutic strategy distinguishes our hydrogel from most existing wound dressings, which primarily target a single pathological aspect. We first characterized the immune microenvironment at day 3 postwound (the peak inflammatory stage) via immunofluorescence staining for pan-macrophage (CD68), M1 (iNOS), and M2 (CD163) markers. The Alg group exhibited high iNOS expression (9.22 ± 1.46%) and low CD163 expression (3.08 ± 0.90%), indicating a pro-inflammatory, M1-dominant microenvironment. Mg-ZIF-8@Alg alone moderately reduced iNOS and increased CD163 expression, TP@Alg alone had a more pronounced effect, and Mg-ZIF-8@TP@Alg exhibited the lowest iNOS expression (3.53 ± 0.57%) and the highest CD163 expression (9.37 ± 1.05%) (Figure 7A,C). These findings confirm that the composite hydrogel exerts a synergistic immunomodulatory effect *in vivo* and can rapidly reverse the pro-inflammatory state that impedes wound healing.

**Figure 7:**
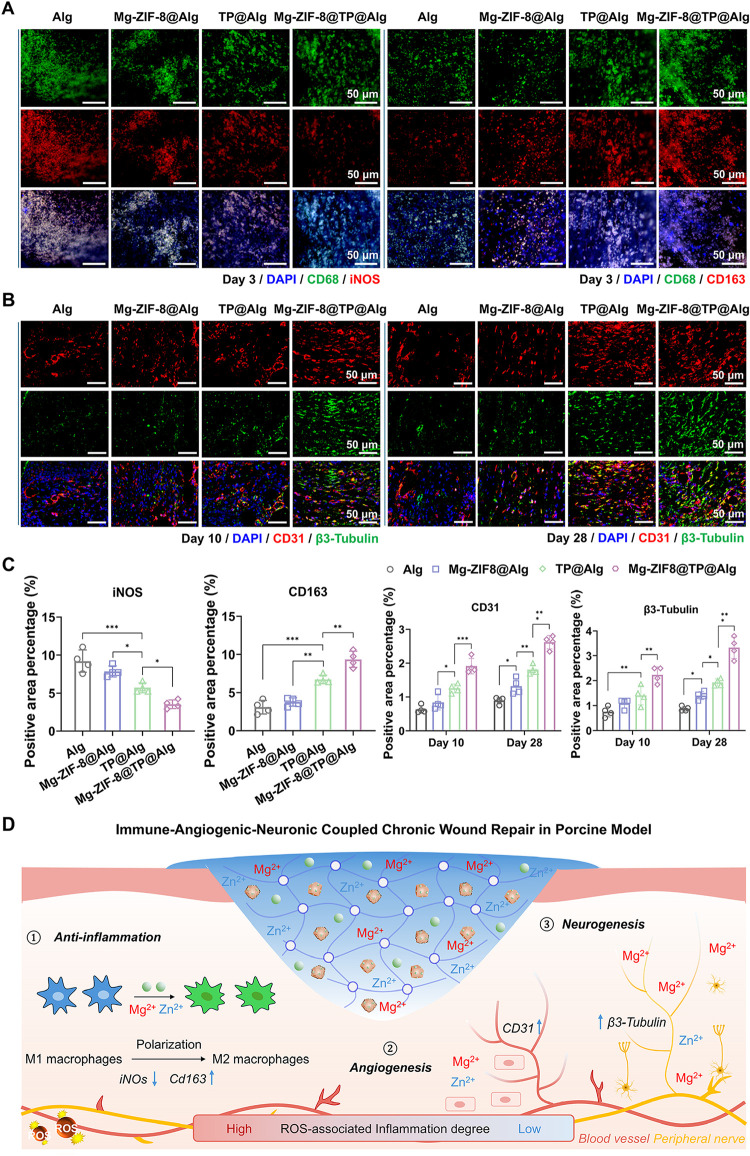
Immune-Angiogenic-Neuronic coupled repair in H_2_O_2_-induced ROS-associated chronic wounds using Mg-ZIF-8@TP@Alg sprayable hydrogel in porcine model. (A) Representative CD68, iNOS, and CD163 IF staining images from the Alg, Mg-ZIF-8@Alg, TP@Alg, and Mg-ZIF-8@TP@Alg treatment groups on 3 days postwound. (B) Representative CD31 and β-3 Tubulin IF staining images from the Alg, Mg-ZIF-8@Alg, TP@Alg, and Mg-ZIF-8@TP@Alg treatment groups on 10 and 28 days postwound. (C) Quantification of iNOS, CD163, CD31, and β-3 Tubulin positive area percentage in Alg, Mg-ZIF-8@Alg, TP@Alg, and Mg-ZIF-8@TP@Alg treated groups (*n* = 4). * *P* < 0.05, ** *P* < 0.01, *** *P* < 0.001, by one or two-way ANOVA with Bonferroni *post hoc* test. (D) Schematic diagram illustrating the mechanism of sprayable Mg-ZIF-8@TP@Alg hydrogel in reversing multifaceted pathology and promoting chronic wound healing in porcine models.

We then evaluated neurovascular regeneration at days 10 and 28 postwound (the proliferation and remodelling stages) via immunofluorescence staining for vascular (CD31) and neuronal (β3-Tubulin) markers. All active treatment groups exhibited increased CD31^+^ vascular density and β3-Tubulin^+^ nerve fiber area compared to the Alg, with a clear efficacy hierarchy: Mg-ZIF-8@TP@Alg > TP@Alg > Mg-ZIF-8@Alg (Figure 7B,C). On day 28, the CD31^+^ vascular density and β3-Tubulin^+^ nerve fiber area in the Mg-ZIF-8@TP@Alg group were 2.62 ± 0.20% and 3.33 ± 0.43%, respectively, significantly higher than those in the other groups. These results confirm that the Mg-ZIF-8@TP@Alg hydrogel can synergistically promote the formation of mature vascular networks and long-range nerve fibers *in vivo*, thereby restoring the immune-angiogenic-neuronic crosstalk required for functional tissue regeneration.^45^

Notably, TP not only exerts antioxidant and anti-inflammatory effects but also promotes neurovascular regeneration by resolving the pro-inflammatory microenvironment that impedes vascular and axonal growth. The sustained release of Mg^2+^ and Zn^2+^ drives direct pro-regenerative effects while further enhancing the anti-inflammatory effect of TP (Figure 7D). This bidirectional synergy and temporal-sequential regulation (TP for early inflammation resolution; Mg^2+^ and Zn^2+^ for late neurovascular regeneration) are key reasons for the superior *in vivo* efficacy of the Mg-ZIF-8@TP@Alg hydrogel, and this coupled immune-angiogenic-neuronic repair mechanism cannot be achieved by conventional wound dressings.

### Transcriptomic Insights into Healing Mechanisms

The molecular mechanisms underlying the sequential therapy of the Mg-ZIF-8@TP@Alg hydrogel were elucidated. To directly validate the early immunomodulatory events, we performed RT-qPCR on wound tissues collected at day 3 (Figure S11). Results indicated that the pro-inflammatory genes *CD86*, *TNF-α*, *iNOS*, and *IL-1β* were significantly downregulated in the TP@Alg-treated group compared to the control and further inhibited by the Mg-ZIF-8@TP@Alg (*P* < 0.001), confirming the effective suppression of the M1 inflammatory response. The anti-inflammatory and M2-associated genes *CD206*, *ARG-1*, *IL-10*, and *IL-4* were significantly upregulated (*P* < 0.01), particularly in the Mg-ZIF-8@TP@Alg-treated group, indicating enhanced M2 macrophage polarization. These data provided direct molecular evidence that, within the anticipated early therapeutic window (Days 1–3), TP and released Mg^2+^, Zn^2+^ indeed orchestrated a shift from a pro-inflammatory to a pro-regenerative immune microenvironment, thereby supporting the core premise of the sequential therapy.

The cumulative therapeutic outcomes (tissue regeneration pathways) were evaluated by bulk RNA sequencing of wound tissues on day 10 postwound. Volcano plots showed the differentially expressed genes (DEGs) between key comparisons (Figure 8A). The analysis revealed that Mg-ZIF-8@Alg treatment upregulated 301 genes and downregulated 291 genes compared to Alg. A more pronounced transcriptional change was observed when comparing Mg-ZIF-8@Alg with ZIF-8@Alg, with 592 upregulated and 766 downregulated genes, highlighting the distinct biological impact of Mg^2+^ doping. The therapeutic effect was most pronounced in the Mg-ZIF-8@TP@Alg group, which showed 863 upregulated and 563 downregulated genes compared with the Alg group. Notably, compared with Mg-ZIF-8@Alg alone, the Mg-ZIF-8@TP@Alg combination altered the expression of 978 genes up and 740 genes down, underscoring the composite’s synergistic regulatory capacity. A Venn diagram further illustrated the respective and overlapping gene sets regulated by each treatment (Figure 8B), and a heatmap of DEGs was shown in Figure S12, confirming the distinct molecular regulatory pattern of Mg-ZIF-8@TP@Alg.

**Figure 8:**
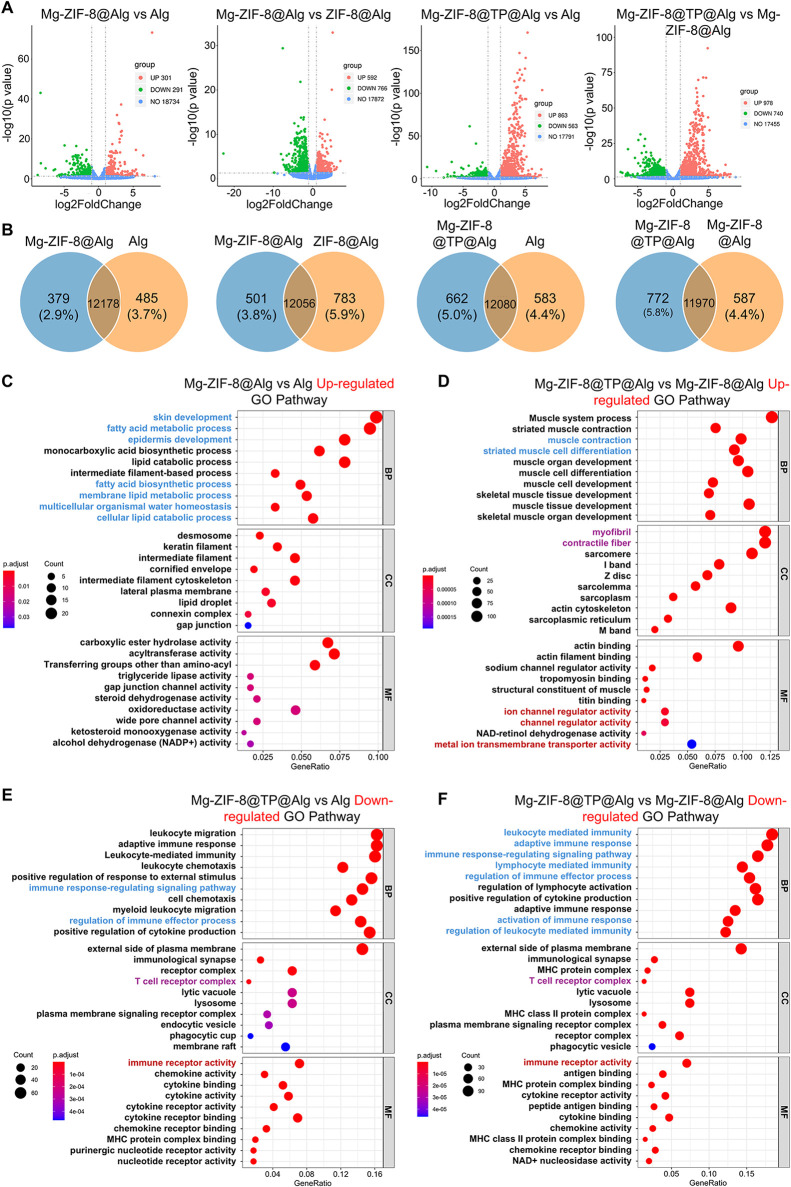
Mechanism of Mg-ZIF-8@TP@Alg hydrogel promoting ROS-associated chronic wound repair. (A) Volcano plots of differentially expressed genes after treating ROS-associated chronic wounds with blank Alg, ZIF-8@Alg, Mg-ZIF-8@Alg, and Mg-ZIF-8@TP@Alg hydrogels for 10 days. (B) Venn diagram of genes after treating ROS-associated chronic wounds with Alg, ZIF-8@Alg, Mg-ZIF-8@Alg, and Mg-ZIF-8@TP@Alg hydrogel for 10 days. (C–D) Up-regulated GO pathway enrichment analysis of differentiated genes among Alg-, Mg-ZIF-8@Alg-, and Mg-ZIF-8@TP@Alg-treated groups. (E-F) Downregulated GO pathway enrichment analysis of differentiated genes among Alg-, Mg-ZIF-8@Alg-, and Mg-ZIF-8@TP@Alg-treated groups.

Gene Ontology (GO) enrichment analysis of upregulated DEGs revealed that Mg-ZIF-8@Alg treatment significantly enriched pathways related to skin and epidermal development, as well as fatty acid and lipid metabolic processes, compared to the Alg group, which is attributed to the bioactive effects of Mg^2+^ and Zn^2+^ on cellular metabolism and skin tissue regeneration (Figure 8C). Mg-ZIF-8@TP@Alg treatment further enriched pathways involved in muscle development, ion channel regulation, and transmembrane transport (Figure 8D), confirming the synergistic effect of TP and bioactive ions (Mg^2+^ and Zn^2+^). Analysis of downregulated DEGs showed that Mg-ZIF-8@Alg alone suppressed pathways associated with immune response regulation and immune receptor activity, whereas Mg-ZIF-8@TP@Alg treatment profoundly downregulated pathways related to leukocyte-mediated immunity, adaptive immune responses, and T-cell receptor activity (Figure 8E,F), a finding consistent with our *in vivo* immunofluorescence results. Gene Set Enrichment Analysis (GSEA) further confirmed significant negative enrichment of B-cell-, T-cell-, and chemokine-mediated immune response gene sets in the Mg-ZIF-8@TP@Alg group (Figure S13), validating the targeted immunosuppressive effect of the composite hydrogel on excessive pro-inflammatory immune responses.

Collectively, the sequential profiles demonstrate that Mg-ZIF-8@TP@Alg hydrogel orchestrates chronic wound repair via a dual molecular mechanism: (1) potent suppression of excessive and detrimental immune activation to resolve chronic inflammation; (2) activation of genetic programs crucial for skin tissue development. This coordinated regulation of immune and regenerative pathways provides a comprehensive molecular basis for the material’s sequential therapeutic effects and confirms that its structural design is closely linked to its molecular regulatory function. Mg^2+^ doping activates regenerative pathways, TP loading suppresses pro-inflammatory pathways, and their combination synergistically regulates the wound microenvironment.

Importantly, we have supplemented the serum ion analysis with histological evaluation of major organs to assess safety. The concentrations of Mg^2+^ and Zn^2+^ in the treated group showed no statistically significant differences from those in the control group (*P* > 0.05), and both remained within normal physiological ranges (Figure S14). H&E staining of the heart, liver, spleen, lung, and kidney revealed intact tissue architecture in all groups, with no evidence of necrosis, inflammation, or other pathological alterations (Figure S15). These data demonstrated that the degradation products of the Mg-ZIF-8@TP@Alg hydrogel did not cause detectable systemic accumulation or toxicity in major metabolic organs.

### Comparison with Existing Research and Study Limitations

This work advances the field of MOF-based wound repair materials in several key aspects: (1) compared to pure ZIF-8, Mg-ZIF-8 endows the material with pro-neurovascular regenerative bioactivity, addressing the intrinsic limitation of ZIF-8 in soft tissue repair. (2) Compared to single Mg-based or TP-based materials, the composite hydrogel achieves sequential therapy and synergistic biofunctions, overcoming the efficacy limitation of single-component materials. (3) Compared to most wound repair studies based on small-animal models (e.g., mice), our large-animal minipig model provides more clinically relevant data, laying a solid foundation for subsequent clinical translation.

Despite these advances, this study has several limitations that warrant future investigation: (1) the efficacy and safety need to be carefully evaluated via clinical trials. (2) The efficacy of the hydrogel in more complex chronic wound models (e.g., diabetic ulcers and ischemic wounds) needs to be verified, as these models better mimic clinical chronic wound pathologies.^46^ Future work will focus on addressing these limitations, scaling up material production, and conducting clinical trials to accelerate the clinical translation of this sprayable hydrogel.

## Conclusion

In this study, we successfully engineered a sprayable Mg-ZIF-8@TP@Alg hydrogel with tailored sequential, coupled immune-angiogenic-neuronic repair properties for treating chronic wounds with complex pathologies. The Mg-ZIF-8 maintains the structural features of ZIF-8, including its pH-responsive degradability and drug-delivery capabilities, while demonstrating enhanced bioactivity. The addition of TP effectively reduces oxidative stress and reprograms macrophage polarization. Mg-ZIF-8@TP achieves different release profiles (TP is rapidly released, whereas bioactive Mg^2+^ and Zn^2+^ are released gradually) and responds to pH changes. It shows excellent *in vitro* biocompatibility, synergistically scavenges ROS, promotes M2 macrophage polarization, and supports neurovascular regeneration. This synergistic approach yields a significantly better therapeutic outcome than single-component treatments, overcoming limitations inherent to either pure TP or unmodified MOFs. In a porcine chronic wound model, the hydrogel treatment markedly accelerated wound closure, modulated the immune microenvironment, increased collagen deposition, and restored the neurovascular network. Molecular analysis further indicates that the hydrogel influences pathways related to skin development, immune responses, and ion transport, thereby orchestrating wound-healing processes. Overall, the Mg-ZIF-8@TP@Alg hydrogel shows strong potential for clinical translation, offering a therapeutic strategy to meet the unmet need for bioactive wound dressings with temporally programmed therapeutic effects.

## Materials and Methods

### Materials

Zinc nitrate hexahydrate (Zn­(NO_3_)_2_·6H_2_O), magnesium nitrate hexahydrate (Mg­(NO_3_)_2_·6H_2_O), 2-methylimidazole (2-MIM), *N*,*N*-dimethylformamide (DMF), sodium alginate, calcium chloride (CaCl_2_), and tea polyphenol (TP, purity ≥ 98%) were purchased from Sinopharm Chemical Reagent Co., Ltd. (Shanghai, China). All reagents were of analytical grade.

### Synthesis of Mg-ZIF-8 Nanoparticles

Mg-ZIF-8 nanoparticles were synthesized via a one-pot method at room temperature. Briefly, 10 mL DMF and 6.0 mmol 2-MIM were mixed for 10 min, followed by the addition of 100 mM Mg­(NO_3_)_2_ and 50 mM Zn­(NO_3_)_2_ solutions (20 mL). The mixture was stirred for 12 h, centrifuged (8000 rpm, 10 min), washed three times with DMF and ethanol, and freeze-dried to obtain Mg-ZIF-8. ZIF-8 was synthesized using the same method without Mg­(NO_3_)_2_·6H_2_O.

### Preparation of Mg-ZIF-8@TP and Mg-ZIF-8@TP@Alg Hydrogel

Mg-ZIF-8@TP was prepared by incubating Mg-ZIF-8 with a TP solution (1:0.5 mass ratio) at room temperature for 3 h, followed by centrifugation and washing. TP loading efficiency was calculated via UV–vis spectroscopy (Thermo Scientific NanoDrop 2000) at 278 nm. Mg-ZIF-8@TP@Alg hydrogel was prepared by mixing Mg-ZIF-8@TP with 2% Alg solution, followed by the addition of 1% CaCl_2_ solution to induce gelation.

### Physical Characterization

The crystal structure of the particles was analyzed using X-ray diffraction (XRD; Bruker D8). Morphology and elemental distribution were observed via transmission electron microscopy (TEM; JEOL JEM-2100) and high-angle annular dark-field (HAADF) scanning TEM. Fourier transform infrared spectroscopy (FTIR; Nicolet iS50) was used to identify functional groups. Particle size and zeta potential were measured by dynamic light scattering (DLS; Malvern Zetasizer Nano ZS90). Atomic force microscopy (AFM; Bruker Dimension Icon) was used to characterize particle thickness. *In vitro* TP, Mg^2+^, and Zn^2+^ release profiles were measured in PBS (pH = 6.0, 7.4, 8.0) at 37 °C. Degradation behavior was observed via TEM after 4 weeks of incubation in PBS. The morphology of the hydrogel was examined using scanning electron microscopy (SEM; JEOL JSM-6360LV). Rheological measurements were performed on preformed hydrogel discs (20 mm diameter, 0.5–1 mm thickness) using a parallel-plate rheometer at 37 °C. Strain sweeps were conducted at 1 Hz to determine the linear viscoelastic region, followed by frequency sweeps (0.1–100 rad/s) at a constant strain of 1%. *In situ* gelation time was conservatively estimated via the inverted vial method on porcine wounds immediately after spraying the Ca^2+^ solution. The practical applicability of the spray-on dressings was visually demonstrated on *ex vivo* porcine skin (Figure S6), confirming their conformability, capacity to fill defects, flexibility, and interfacial adhesion stability.

### *In Vitro* Cell Culture

RAW 264.7 macrophages and HUVECs were cultured in DMEM supplemented with 10% fetal bovine serum (FBS) and 1% penicillin-streptomycin at 37 °C in 5% CO_2_.

### Cytocompatibility Assay

Cells were seeded in 96-well plates (5 × 10^3^ cells/well) and treated with different concentrations of Mg-ZIF-8 (0, 0.1, 1, 10, 25, 50 μg/mL) for 1–3 days. Cell viability was evaluated via CCK-8 assay. Live/dead staining was performed using Calcein-AM and Propidium Iodide (PI) to visualize cell survival.

### Antioxidant Activity Assay

Cells were pretreated with H_2_O_2_ (100 μM) for 2 h to induce oxidative stress, then treated with Mg-ZIF-8 (10 μg/mL), TP (3 μg/mL), or Mg-ZIF-8@TP (13 μg/mL) for 24 h. Intracellular ROS levels were detected using the DCFH-DA fluorescent probe and analyzed via fluorescence microscopy. Here, the TP amount was calculated based on the drug-loading efficiency (30%), and Mg-ZIF-8@TP denotes the total amount of Mg-ZIF-8 and TP.

### Immunomodulation Assay

RAW 264.7 macrophages were treated as above, then immunofluorescently stained for iNOS, CD86 (M1 marker), and CD163 (M2 marker).

### Angiogenic Activity Assay

HUVECs were treated with normal medium or a medium containing Mg-ZIF-8, TP, and Mg-ZIF-8@TP following H_2_O_2_ induction. Cell migration was evaluated using a scratch assay (18 h). The tube formation assay was performed on Matrigel-coated plates, with tube meshes and branches quantified using ImageJ.

### Peripheral Nerves Regeneration Assay

According to prior studies, primary Schwann cells were isolated from 2-week-old SD rats, and DRG neurons were isolated from GFP transgenic rats.^47^ Schwann cells and DRG neurons were treated with PBS, Mg-ZIF-8, TP, or Mg-ZIF-8@TP-containing medium after H_2_O_2_ induction. Schwann cell migration was evaluated using a transwell assay (24 h). Myelination was evaluated 2 days post-treatment by immunofluorescent (IF) staining with primary antibodies against β-III tubulin (ab6161, Abcam) and GAP-43 (ab16053, Abcam). Randomly selected neurons were captured and analyzed for axonal regeneration using ImageJ after DRG neurons were treated for 3 days.

### *In Vivo* Chronic Wound Model and Treatment

All animal experiments were performed in accordance with protocols evaluated and approved by the Shenzhen Advanced Animal Study Service Center (Ethics Approval Number: AMS F2408022P). Considering the model stability, long induction periods, high failure rates, and considerable cost of the diabetic pig wound model, we adopted the H_2_O_2_-induced chronic wound model to stimulate sustained oxidative stress and impaired healing. Full-thickness skin wounds (1 cm × 2 cm) were created on the back of minipigs using a surgical blade. H_2_O_2_ (100 μL, 100 mM) was injected into the wound bed to induce chronic inflammatory wounds. Wounds were randomly divided into four groups (*n* = 4 per group): Ctrl (Pure Alg), Mg-ZIF-8@Alg, TP@Alg, and Mg-ZIF-8@TP@Alg. Prefabricated hydrogels (∼1 mL) were applied directly to wounds rather than sprayed to ensure precise dosage control, thereby minimizing variability in quantitative analyses. Wound images were captured on days 0, 3, 10, and 28, and the wound area was quantified using ImageJ.

### Histological and Immunofluorescence Analysis

Wound tissues were collected at the study end point, fixed in 4% paraformaldehyde, embedded in paraffin, and sectioned (10 μm). Hematoxylin and Eosin (H&E) staining was conducted to evaluate wound closure and biosafety of main organs, while Masson’s trichrome staining was performed to assess collagen deposition. Immunofluorescence staining was conducted for CD68 (macrophage marker), iNOS (M1 marker), CD163 (M2 marker), CD31 (endothelial cell marker), and β3 tubulin (peripheral nerve marker). Positive areas were quantified via ImageJ.

### RNA Sequencing and Bioinformatics Analysis

To directly validate the early immunomodulatory events, RT-qPCR was performed on wound tissues collected at Day 3 following the manufacturer’s instructions (Takara Bio Inc., USA). For bulk RNA-seq, total RNA was extracted from wound tissues (day 10) using TRIzol reagent. RNA sequencing was performed on an Illumina NovaSeq 6000 platform. Differentially expressed genes (DEGs) were identified with |log2FC| > 1 and *P* < 0.05. Gene Ontology (GO) enrichment and Gene Set Enrichment Analysis (GSEA) were performed to elucidate biological pathways.

### Statistical Analysis

Data were expressed as mean ± standard deviation. Statistical analysis was performed using GraphPad Prism 9.0. One-way or two-way ANOVA, with appropriate *post hoc* tests, was used for multiple comparisons. *P* < 0.05 was considered statistically significant, with * *P* < 0.05, ** *P* < 0.01, *** *P* < 0.001. Details of the sample size n were provided in each figure legend.

## Supplementary Material



## Notes

Data on gelation time (Table S1), AFM morphology (Figure S1), standard curves (Figure S2), drug loading efficiency (Figure S3), degradation and ion release profiles (Figure S4), rheological properties of hydrogels (Figure S5), key application performances of spray-on dressings (Figure S6), tube formation assays (Figures S7–S9), Schwann cell immunofluorescence (Figure S10), quantitative real-time PCR (Figure S11), gene expression heatmap (Figure S12), GSEA plots (Figure S13), serum ion analysis (Figure S14), and histological staining of major organs (Figure S15) (PDF)
