# Supplementary material for: An Innovative Sprayable Mg-MOF Hydrogel Enhances Healing of Chronic Wounds with Multifaceted Pathologies in a Porcine Model
Source: ACS Nano. 2026 Jul 29;20(31):21921–38. doi: 10.1021/acsnano.6c05950 (PMC13472324; doi:10.1021/acsnano.6c05950)
Supplement: Supplementary file 1 [file nn6c05950_si_001.pdf]

Supporting Information

An Innovative Sprayable Mg-MOF Hydrogel Enhances Healing of Chronic Wounds with Multifaceted Pathologies in a Porcine Model

Shunxiang Xu[1,2,#], Kexin Zhang[3,#], Hongwei Shao[1,2], Qinyu Tian[1,2], Yuantao Zhang[1,2], Jiankun Xu[1,2], Congqin Ning[3,*], Ling Qin[1,2,*]

1 Musculoskeletal Research Laboratory, Department of Orthopaedics & Traumatology, Faculty of Medicine, The Chinese University of Hong Kong, Hong Kong SAR 999077, P. R. China

2 Innovative Orthopaedic Biomaterial and Drug Translational Research Laboratory, Li Ka Shing Institute of Health Sciences, Prince of Wales Hospital, The Chinese University of Hong Kong, Hong Kong SAR 999077, P. R. China

3 The Education Ministry Key Lab of Resource Chemistry and Shanghai Frontiers Science Center of Biomimetic Catalysis and Shanghai Engineering Research Center of Green Energy Chemical Engineering, Shanghai Normal University, Shanghai 200234, P. R. China

*Correspondence authors:

Congqin Ning (cqning@shnu.edu.cn); Ling Qin (lingqin@cuhk.edu.hk).

Shunxiang Xu and Kexin Zhang contributed equally to this work.

Table S1. ***In situ*** **gelation time measurements**

Group	Alg	Mg-ZIF-8@Alg	Mg-ZIF-8@TP@Alg
In situ gelation time (s)	< 5s	< 5s	< 5s

Note: Due to the instantaneous nature of the ionic crosslinking between alginate and Ca^{2+}, obtaining precise second-level timings for the gelation process via manual mixing was unattainable. Therefore, a conservative estimate was obtained using the inverted vial method on porcine wounds after spraying the Ca^{2+} solution, revealing an in situ gelation time of <5 s for all groups.

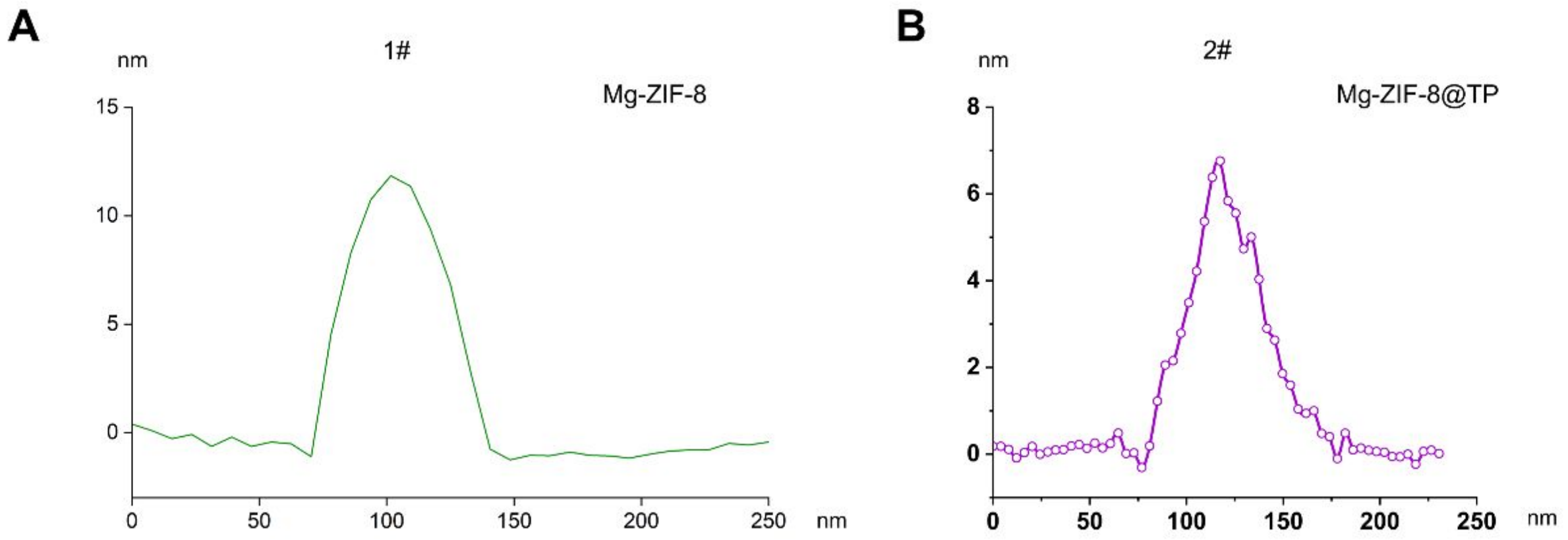

Figure S1. AFM typical height profile in a 2D view of (A) Mg-ZIF-8 (1#) and (B) Mg-ZIF-8@TP (2#) nanoparticles.

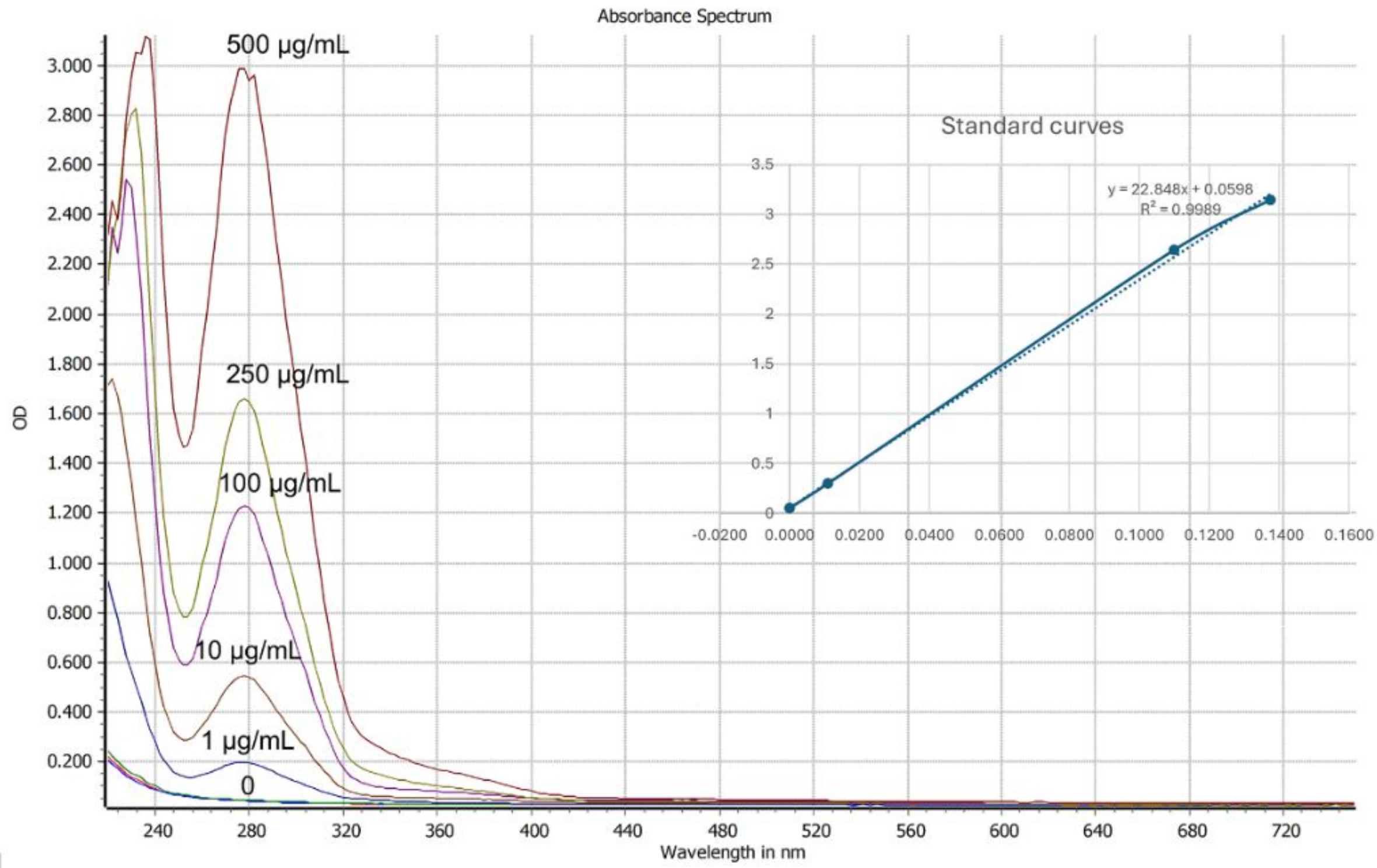

Figure S2. Standard curves of TP solutions.

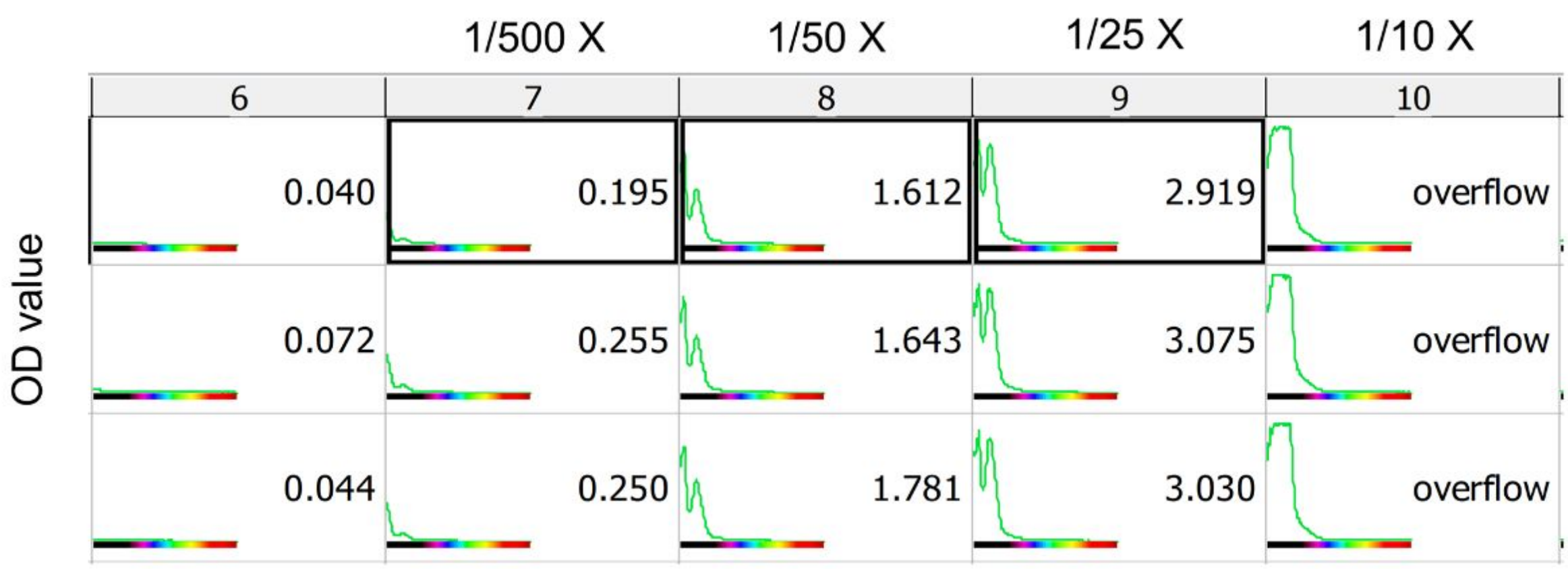

Figure S3. OD value and absorbance spectrum of the supernatant after drug loading.

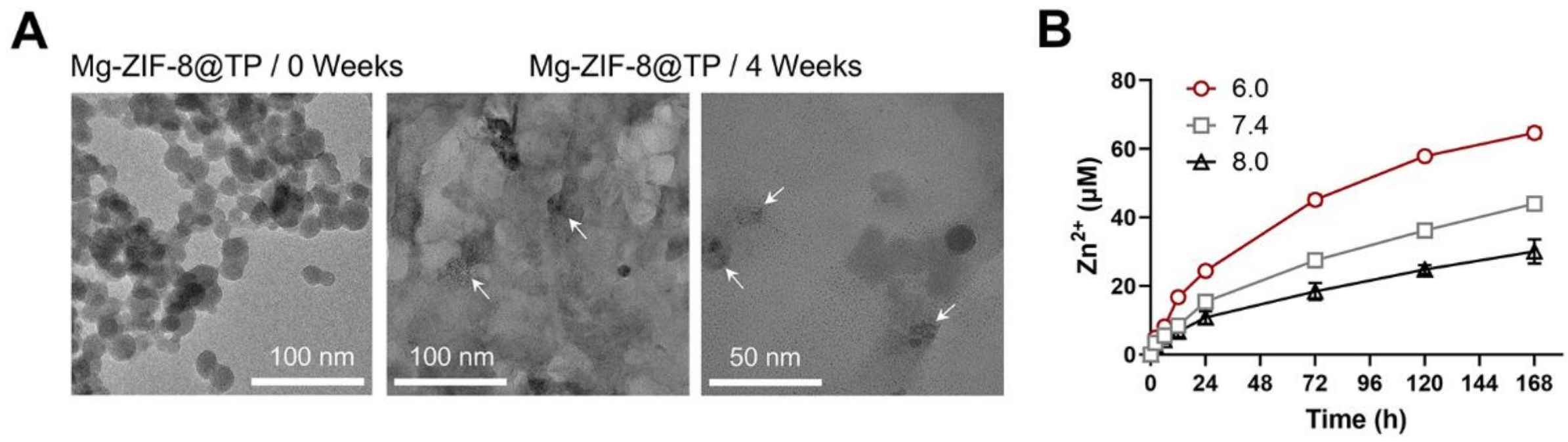

Figure S4. Degradation and ion release. (A) Representative TEM images of Mg-ZIF-8@TP before and after 4 weeks of degradation in PBS solutions. (B) Cumulative release of Zn^{2+} from Mg-ZIF-8@TP nanoparticles under different pH conditions (pH = 6.0, 7.4, 8.0).

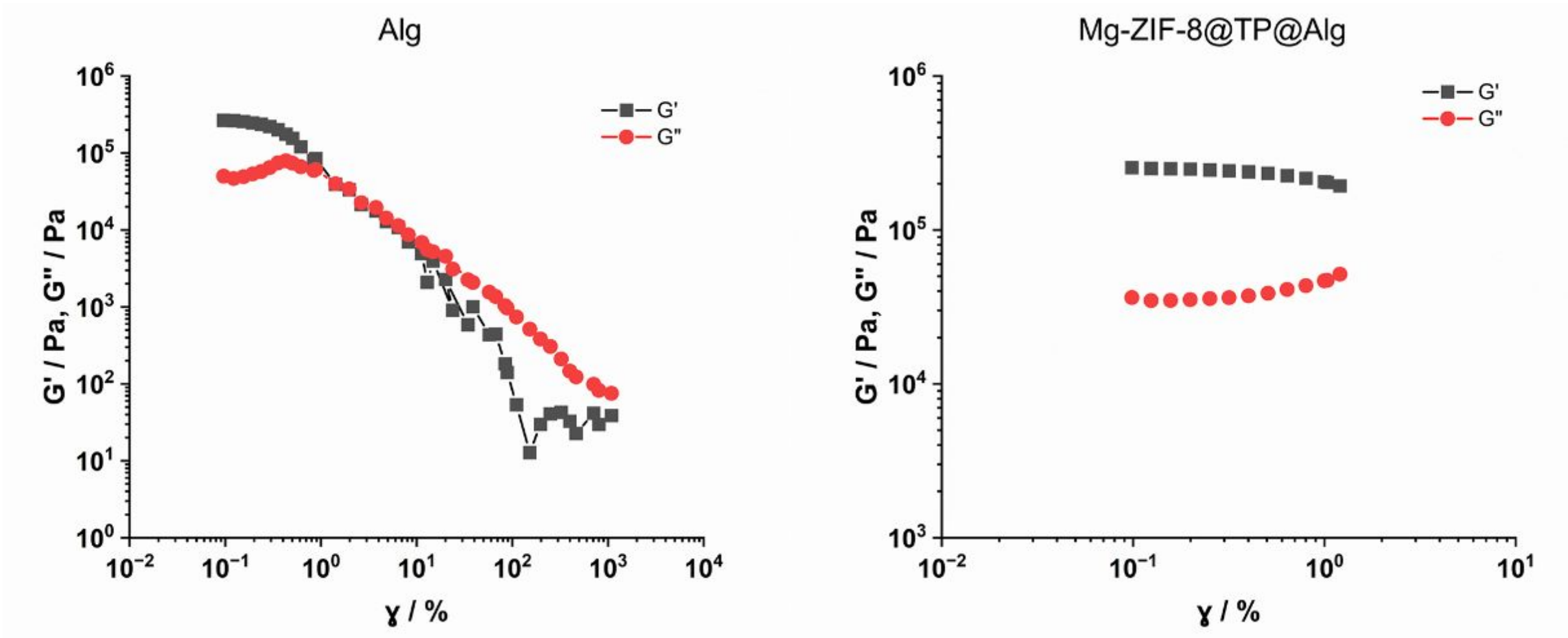

Figure S5. Strain-sweep curves for the storage modulus (G', black squares) and loss modulus (G'', red circles) of Alg and Mg-ZIF-8@TP@Alg hydrogels.

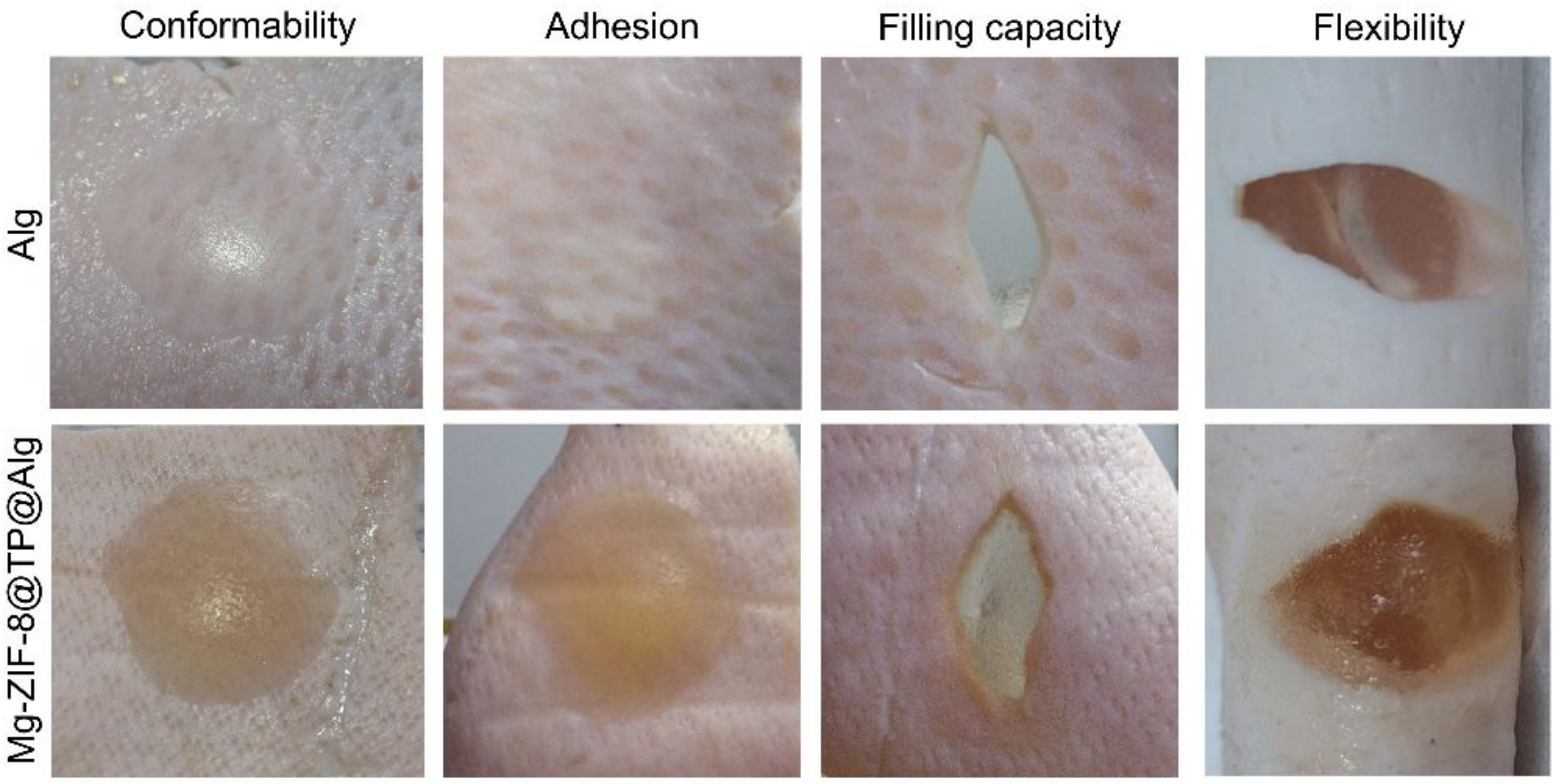

Figure S6. Demonstration of the key application performances of two spray-on dressings. Conformability, adhesion, filling capacity, and flexibility of Alg and Mg-ZIF-8@TP@Alg hydrogels on ex vivo porcine skin.

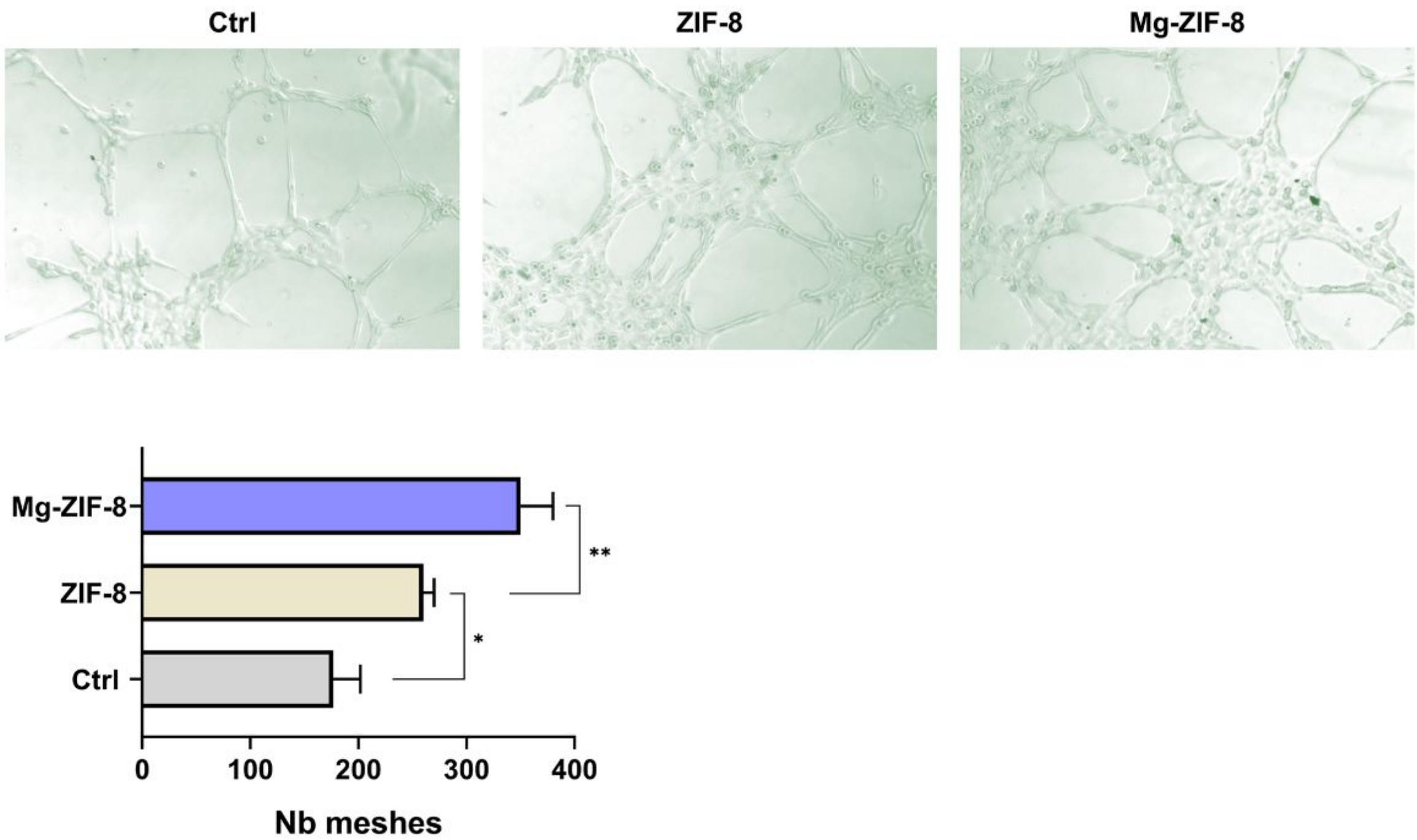

Figure S7. Representative images of tube formation and statistical results. * P < 0.05, ** P < 0.01, by one-way ANOVA with Bonferroni *post hoc* test.

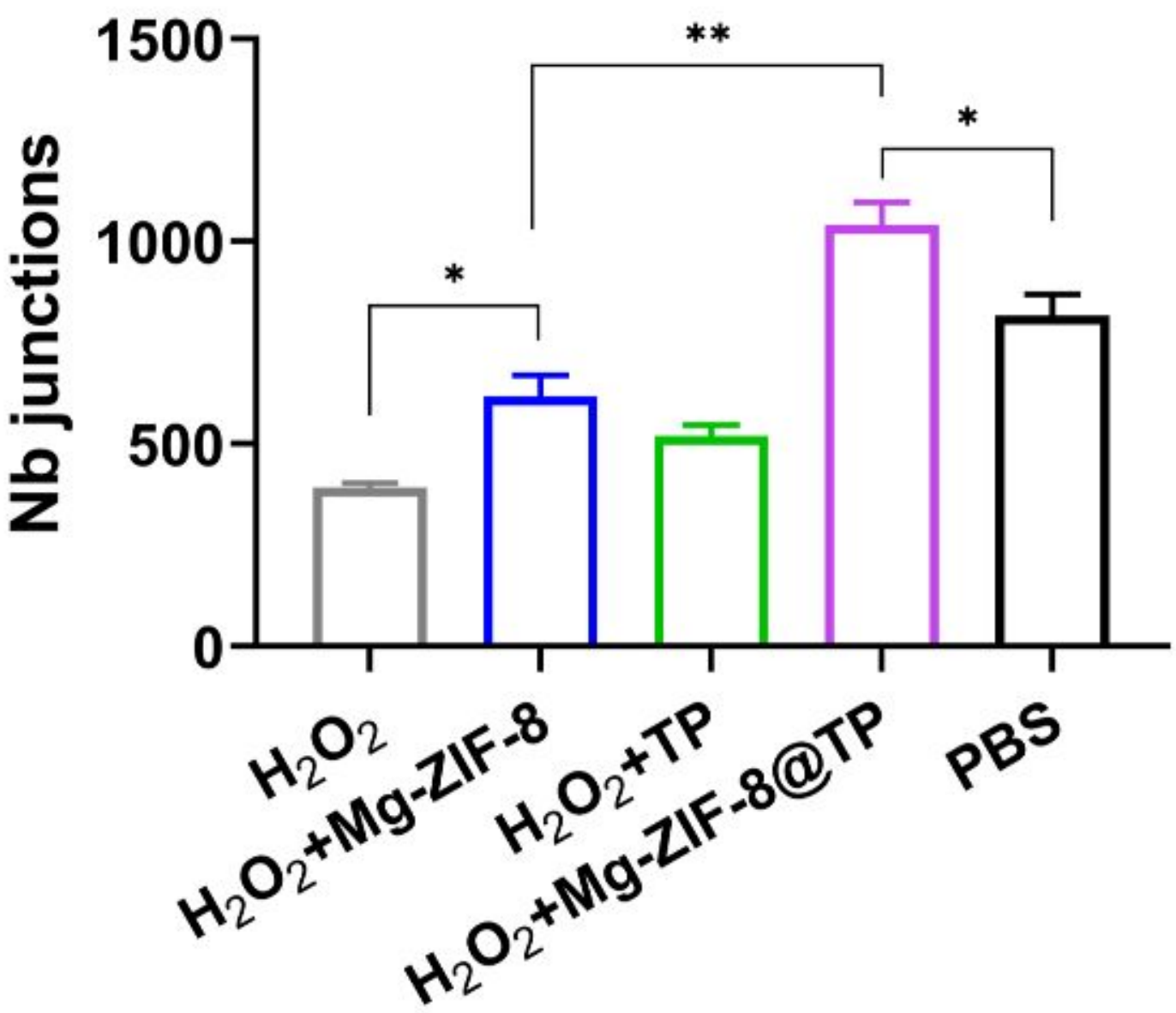

Figure S8. Statistical results of Nb junctions. * P < 0.05, ** P < 0.01, by one-way ANOVA with Bonferroni *post hoc* test.

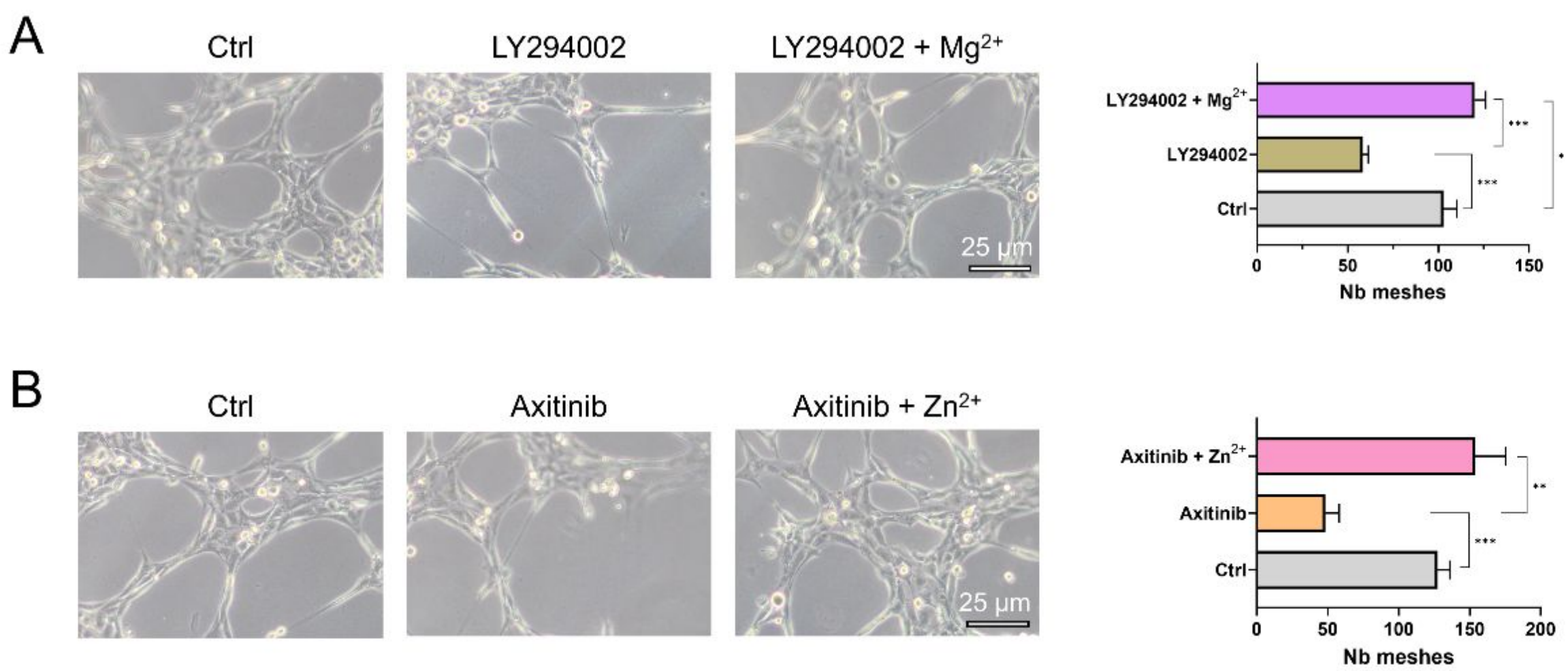

Figure S9. Representative images of tube formation and statistical results. (A) HUVECs treated with Ctrl, PI3K inhibitor LY294002, and LY294002 + Mg^{2+}. (B) HUVECs treated with

Ctrl, VEGFR inhibitor Axitinib, and Axitinib + Zn^{2+}. * $P < 0.05$, ** $P < 0.01$, *** $P < 0.001$, by one-way ANOVA with Bonferroni *post hoc* test.

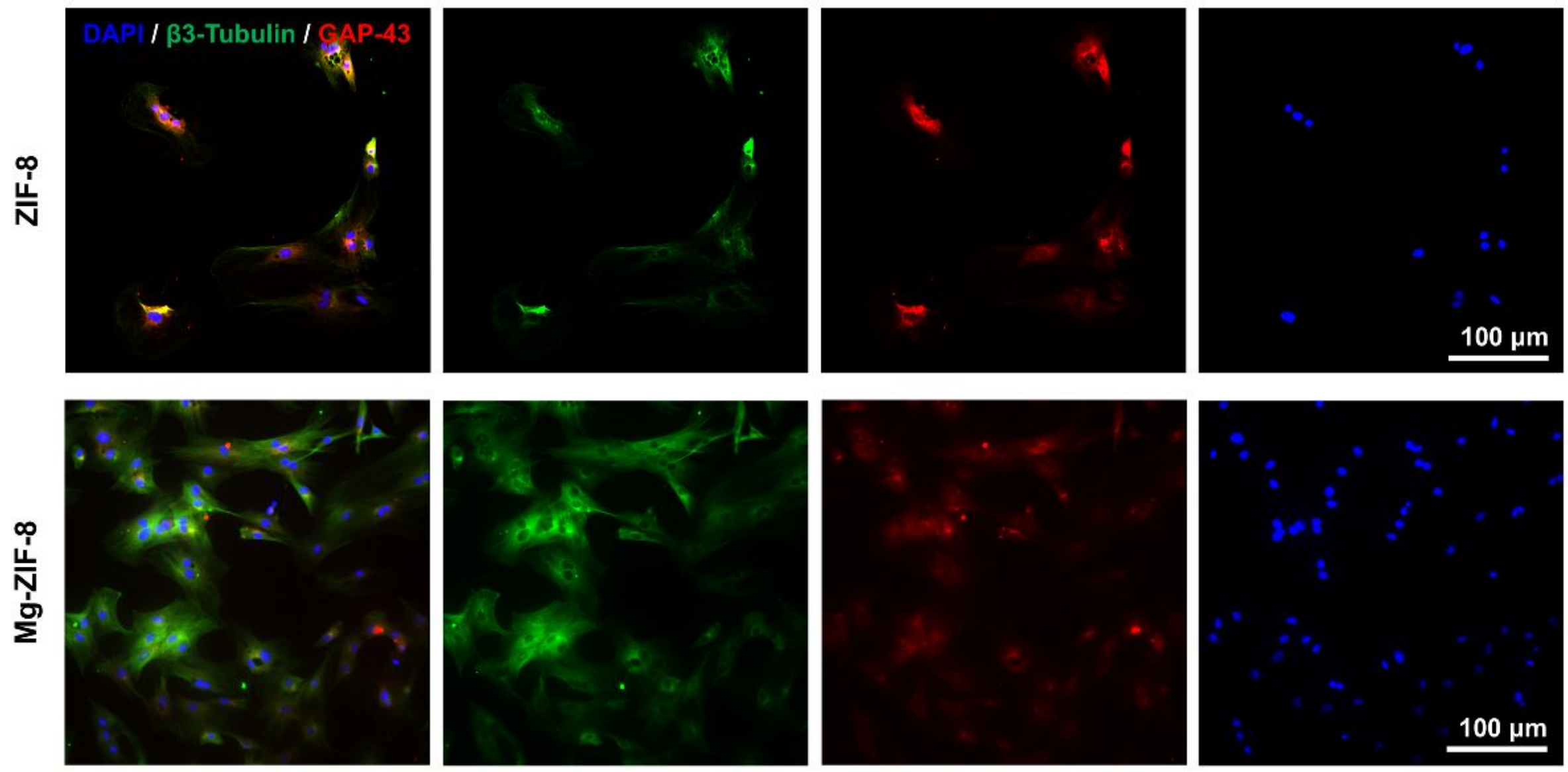

Figure S10. Representative IF images of Schwann cells under the stimulation of ZIF-8 and Mg-ZIF-8 for 48 h (Green, β-3 Tubulin; Red, GAP-43; Blue, DAPI).

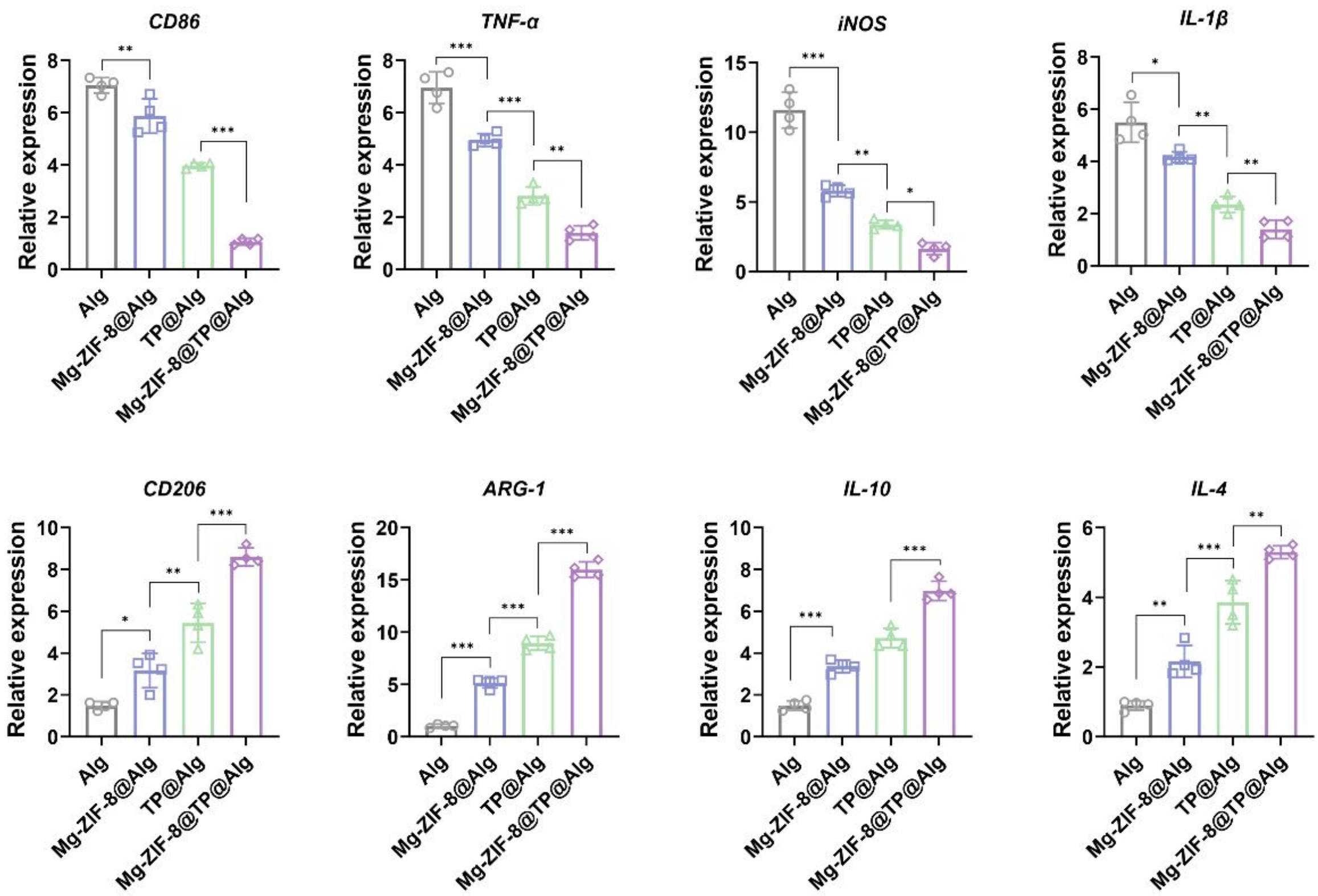

Figure S11. TR-qPCT. Differentiated gene expression after treating ROS-associated chronic wounds with a sprayable hydrogel comprising Alg, Mg-ZIF-8@Alg, TP@Alg, and Mg-ZIF-8@TP@Alg for 3 days.

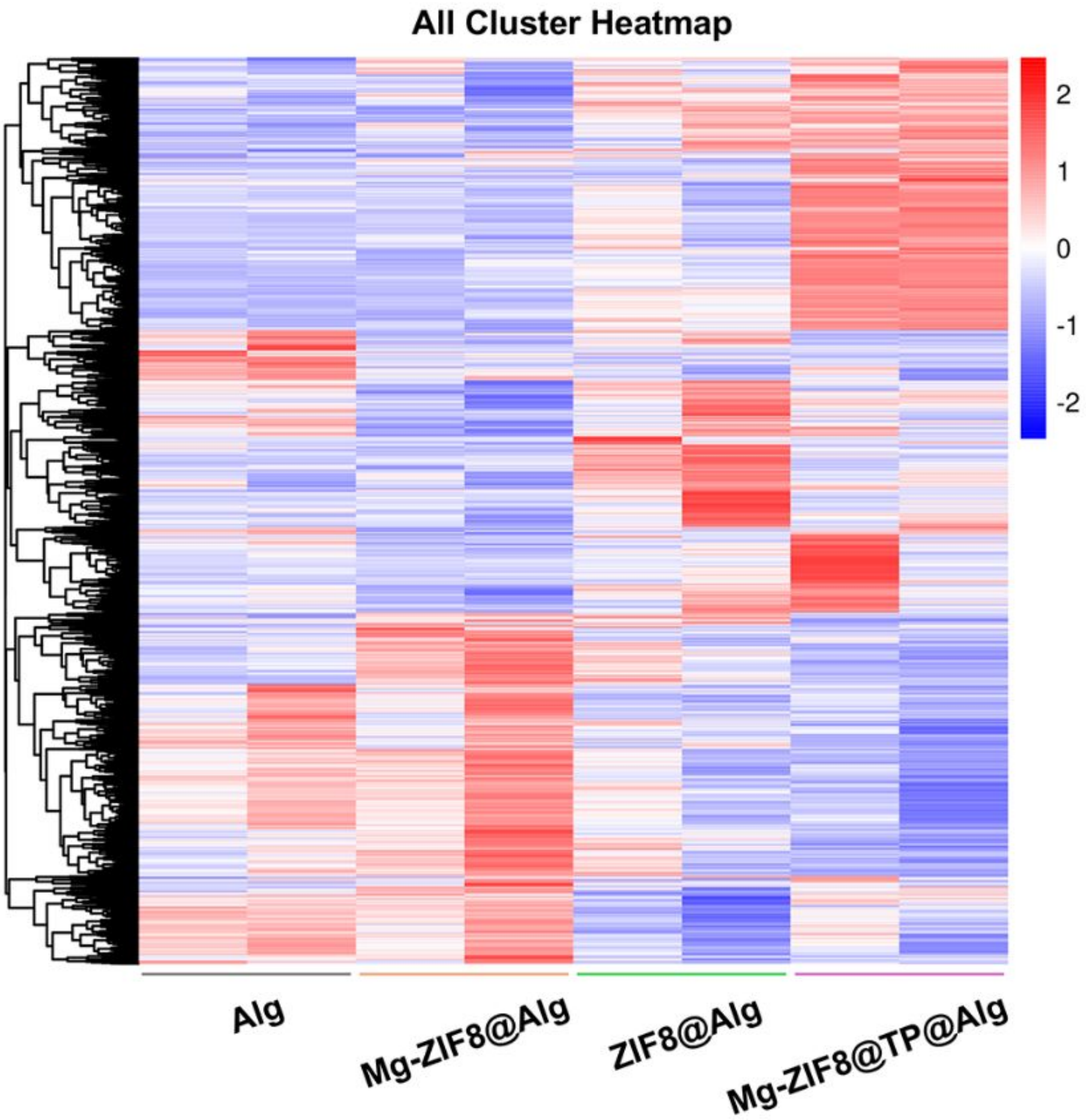

Figure S12. Heatmap of differentiated genes after treating ROS-associated chronic wounds with Alg, Mg-ZIF-8@Alg, ZIF-8@Alg, and Mg-ZIF-8@TP@Alg sprayable hydrogel for 10 days.

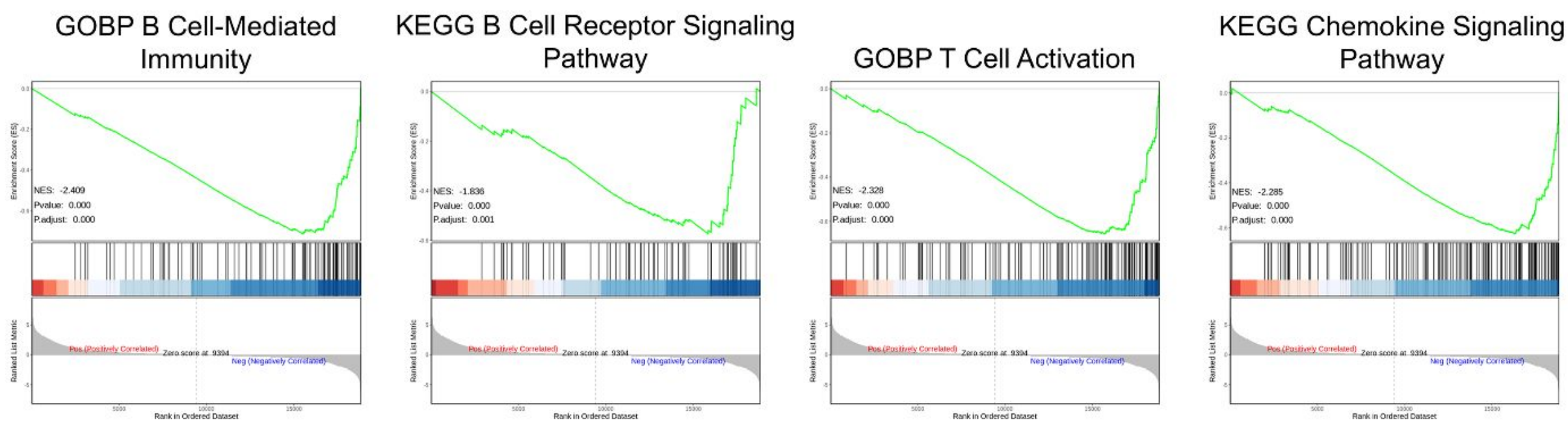

Figure S13. GSEA plots in Mg-ZIF-8@TP@Alg treated groups.

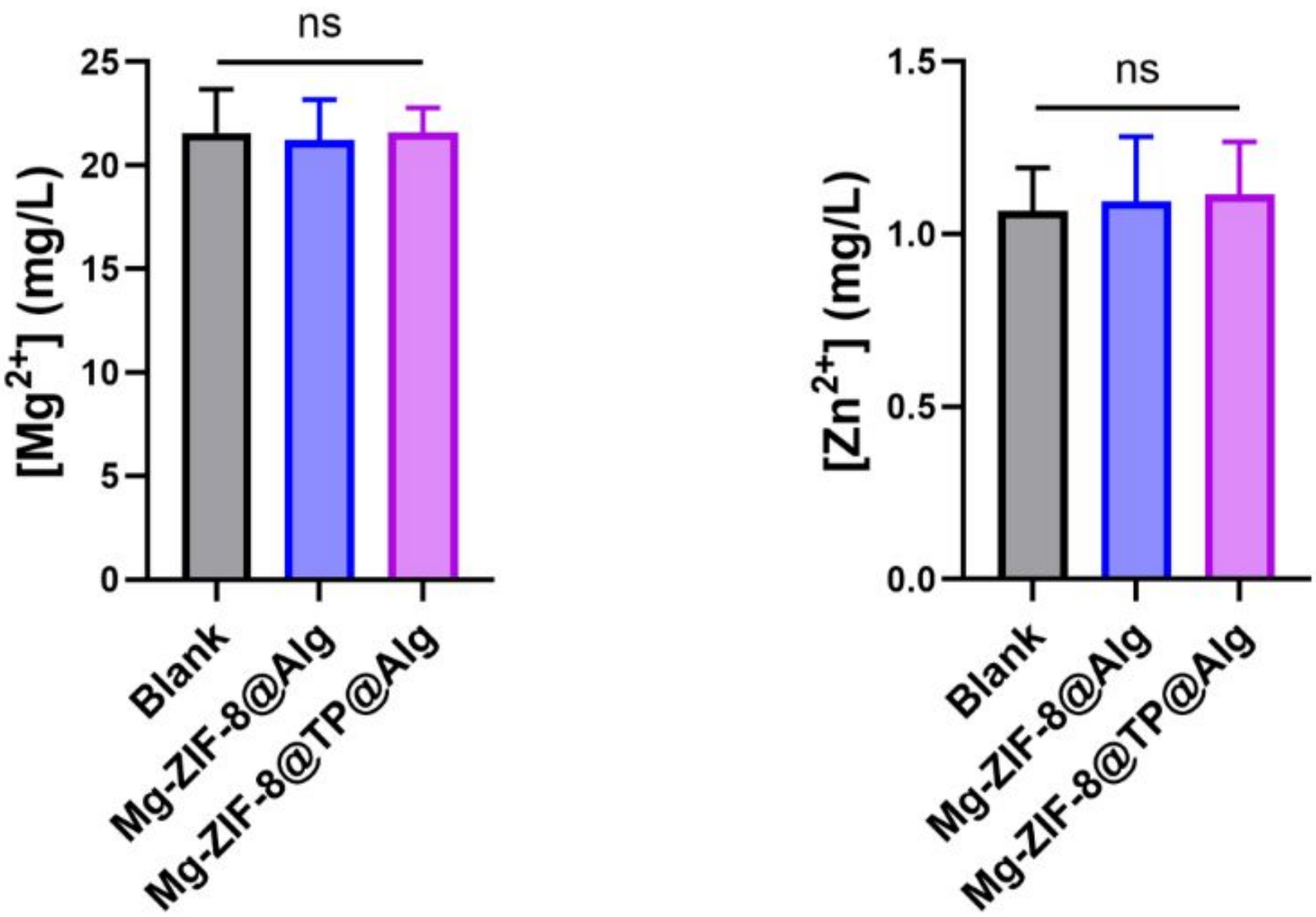

Figure S14. Serum ion analysis of Mg^{2+} and Zn^{2+} after 28 days of treatment with different hydrogels.

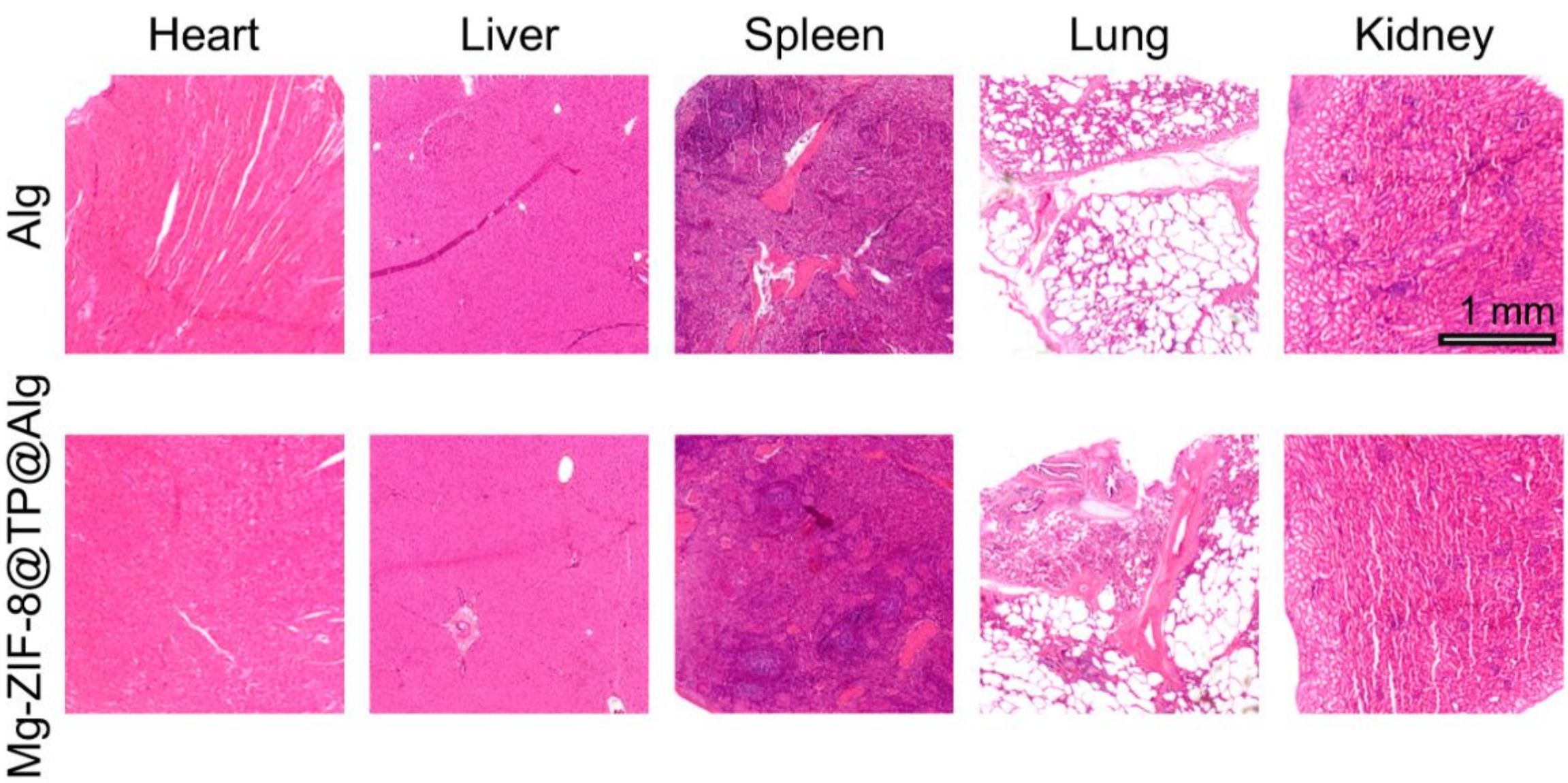

Figure S15. H&E staining of the major organs (heart, liver, spleen, lung, and kidney) collected on 28 days post treatment.
